# Adequate levels of dietary sulphur amino acids impart improved liver and gut health in juvenile yellowtail kingfish (*Seriola lalandi*)

**DOI:** 10.1017/S0007114522002458

**Published:** 2023-04-28

**Authors:** Caroline Lourdes Candebat, Frances Stephens, Mark A. Booth, Fernando Fernando, Andreas Lopata, Igor Pirozzi

**Affiliations:** 1 Centre for Sustainable Tropical Fisheries and Aquaculture & College of Science and Engineering, James Cook University, Townsville, QLD, Australia; 2 Consultant Fish Pathologist, Department of Fisheries, Perth, WA, Australia; 3 NSW Department of Primary Industries, Port Stephens Fisheries Institute, Taylors Beach, NSW 2316, Australia; 4 Australian Institute of Tropical Health and Medicine, James Cook University, Townsville, QLD, Australia; 5 Tropical Futures Institute, James Cook University, Singapore, Singapore

**Keywords:** Aquaculture, Yellowtail kingfish, Methionine, Taurine, Posterior intestine histology, Liver histology, Goblet cells

## Abstract

The sulphur amino acids methionine (Met) and cysteine (Cys) and their derivative taurine (Tau) are metabolically active molecules with interlinked roles in nutritional requirements. Deficiencies in these nutrients are linked to poor growth and health; however, the impacts of these deficiencies on organ structure and function are largely unknown. This study examined the effects of dietary Met, Cys and Tau fed at different levels on yellowtail kingfish (YTK) liver histology and surface colour, plasma biochemistry and posterior intestine histology. Samples were collected from two dose–response feeding trials that quantified (1) the Tau requirement and sparing effect of Met by feeding YTK diets containing one of seven levels of Tau at one of two levels of Met and (2) the Met requirement and sparing effect of Cys by feeding YTK diets containing one of five levels of Met at one of two levels of Cys. YTK fed inadequate levels of dietary Met, Cys and Tau exhibited thicker bile ducts, less red livers, more intestinal acidic goblet cell mucus and supranuclear vacuoles and less posterior intestinal absorptive surface area. Further, thicker bile ducts correlated with less red livers (a*, R), whereas increased hepatic fat correlated with a liver yellowing (b*). Our results indicate a shift towards histological properties and functions indicative of improved intrahepatic biliary condition, posterior intestinal nutrient absorption and homoeostasis of YTK fed adequate amounts of Met, Cys and Tau. These findings may assist in formulating aquafeed for optimised gastrointestinal and liver functions and maintaining good health in YTK.

Fishmeal in aquafeed formulations for carnivorous fish can be progressively replaced using a nutrient-based approach in which dietary proteins that may lack essential nutrients are balanced with crystalline and synthetic nutrients to meet the dietary requirements of the species. Although these proteins are more sustainable and economically viable, the efficient production of healthy fish requires the quantification of the obligatory dietary requirements and measurements on the capacity for metabolic bioconversions in target species. Further, nutrient-based formulations carry the risk of over- or undersupplying essential dietary nutrients, especially when the species-specific nutrient requirements are unknown.

Yellowtail kingfish (*Seriola lalandi*; hereafter referred to as YTK) is a carnivorous fish farmed for its high-quality white meat^(1)^ and increased demands, in tandem with the limited supply of marketable quantities, have led to an increased interest in YTK feeds. Previous research has demonstrated inferior growth, feed efficiency and pathological conditions in YTK fed sub- and supra-optimal levels of the sulphur amino acids (SAA’s) methionine (Met), cysteine (Cys) and also taurine (Tau) in low-fishmeal diets. Such conditions included small white spots and green patches on the liver, cataracts and enteritis^(2–4)^.

In fish, Met deficiencies are associated with an increase in retinal degeneration, bilateral cataract, non-infectious enteritis, inflammatory cell invasion, widening of the lamina propria and hepatocyte necrosis and atrophy^(5)^, whereas dietary Met exceeding physiological requirements leads to histopathological lesions in intestines and livers^(6)^, and inferior intestinal microbiota richness, diversity^(7)^ and performance^(8)^. In fish, 39–60 % of absorbed dietary Met is transsulphurated to endogenous Cys when fed adequate amounts of dietary Met; 39–60 % is also the proportion of dietary Cys that can spare the total SAA (TSAA) requirement for the transsulphuration of Met to Cys in various fish species^(2,9–11)^. Although Cys is a semi-essential amino acid in aquafeeds, the intracellular availability of Cys remains critical for the fitness of animals, forming substrate for the *de novo* synthesis of glutathione, pyruvate, inorganic sulphur, CoA and Tau^(12–14)^. However, excess dietary Cys impairs growth, while excess injected Cys in fish causes mortality in a manner that is similar to excess in Met^(15,16)^. A high level of Cys has also been shown to induce pathological liver conditions in rats^(17,18)^.

In animals, the hepatic transsulphuration and decarboxylation of Met and Cys produce Tau, an amino sulphonic acid, which is considered semi-essential in fish^(19,20)^. In fish, Tau contributes to the maintenance of physiological homoeostasis by (1) conjugating with bile acid, emulsifying ingested lipids in the intestine^(21)^; (2) conjugating with bilirubin to form ditaurobilirubin, eliminating the toxic by-products of heme breakdown through bile^(22,23)^; (3) regulating glucose metabolism^(24)^ and (4) osmoregulating and stabilising the membranes of erythrocytes^(25)^. Tau supplementation to a plant protein-based diet ameliorated gut inflammatory markers and apoptosis in the European seabass intestine^(26)^ and reduced the signs of green liver syndrome in Japanese yellowtail (*S. quinqueradiata*) fed a zero-fishmeal diet^(27)^. Both studies indicated beneficial effects of Tau in fish; however, information on single and interactive effects of dietary Met, Cys and Tau on fish intestinal and hepatic health is still limited^(5,28,29)^.

Candebat *et al.* ^(19)^ established that the Tau requirement in YTK can be satisfied by sufficient dietary Met, inferring that Tau is conditionally dispensable. Follow-up experimentation further quantified the minimum obligatory Met requirement, the TSAA requirement and Cys sparing capacity for Met to meet the TSAA requirement in juvenile YTK^(2)^. YTK fed sub- and supra-optimal levels of SAA exhibited poor feed conversion ratios and specific growth rates, but it was only at sub-optimal levels of SAA that YTK developed clinical signs of cataracts^(2)^. The dietary Met, Cys and Tau appear to play critical roles in the metabolic pathways of carnivorous fish to maintain good health, sustained growth and resistance to environmental stressors, including pathogens^(20)^. Nevertheless, knowledge of the effects of dietary Met, Cys and Tau on clinical pathology and adverse posterior intestinal and hepatic changes are, to our knowledge, limited in YTK.

Overall, deficiencies in essential amino acids are recognised to impair growth in fish, yet knowledge on the extensiveness of their physiological functions and metabolic interrelatedness are still being investigated. Histomorphometric and histochemical measurements of fish organs and tissues can elucidate the modifying effects of dietary amino acids on inflammatory, immune, digestive, homoeostatic stability, compositional and metabolic functions^(5,30,31)^. A comprehensive quantitative histological and biochemical dataset will provide reference values for fish and would serve not only as a diagnostic tool for clinical symptoms but also as a biomarker to identify adequate dietary level of SAA that may optimise physiological and histological conditions.

The aim of this study is to observe the homoeostatic, metabolic, protective, digestive and absorptive properties of the systems to evaluate the health status of YTK, derived from the requirement studies published by Candebat *et al.* ^(2,19)^, and determine the extent to which replete, deficient or in excess combinations of dietary Met, Cys and Tau may alter function.

## Materials and methods

### Ethics statement

All experiments were performed under the NSW DPI Fisheries Animal Care and Ethics Research Authority, known as ‘Aquaculture Nutrition ACEC 93/5–Port Stephens’^(32)^, and were conducted at the Port Stephens Fisheries Institute, NSW, Australia.

### Experimental design

Samples from two separately conducted feeding trials^(2,19)^ were collected to investigate the effects of different dietary Met, Cys and Tau levels on liver histology, posterior intestine histology, blood plasma biochemistry and liver surface colour in juvenile YTK. Detailed descriptions of experimental design, diet formulation and manufacture, experimental facilities and procedures are presented in Candebat *et al.* ^(19)^ and Candebat *et al.* ^(2)^.

Throughout the present study, the first feeding trial by Candebat *et al.* ^(19)^ will be referred to as TauMet study and diets annotated with T + M. Briefly, the objective of the TauMet study was to quantify the Tau requirement in juvenile YTK at a Met content that either met or exceeded current industry practice (11 g Met/kg diet)^(19)^. For that purpose, a factorial dose–response approach was applied, using seven incremental levels of Tau, ranging from 1·6 to 20·4 g/kg diet, crossed with either one of two levels of dietary Met (10·9 g/kg diet or 17·2 g/kg diet) at one constant Cys level (mean: 5·9 (sem 0·2) g/kg diet; Table 1), resulting in fourteen experimental diets (T + M 1–14). Each diet was randomly allocated to three replicated 200 l tanks, each stocked with fourteen fish (initial body weight; 53·3 (sem 0·4) g/fish). After 7 weeks, tissue samples from fish (*n* ≥ 6) from all fourteen feeding treatments were collected, all of which were analysed for liver surface colour and plasma biochemical analysis, and of which selected dietary treatments of YTK were analysed for liver and posterior intestine histology. Please refer to Table 2 for further information on selected dietary treatments and abbreviations.

**Table 1.** Formulation and composition of the TauMet experimental diets

	Low methionine series							High methionine series						
	T + M 1	T + M 2	T + M 3	T + M 4	T + M 5	T + M 6	T + M 7	T + M 8	T + M 9	T + M 10	T + M 11	T + M 12	T + M 13	T + M 14
Raw material composition (% DM basis)*														
Wheat flour	17·9	16·2	15·4	15·4	15·4	15·4	15·4	15·4	15·4	15·4	15·4	15·4	15·4	15·4
Poultry meal	12·0	12·0	12·0	12·0	12·0	12·0	12·0	12·0	12·0	12·0	12·0	12·0	12·0	12·0
Fish oil	11·0	11·0	11·0	11·0	11·0	11·0	11·0	11·0	11·0	11·0	11·0	11·0	11·0	11·0
Dehulled lupin	10·0	10·0	10·0	10·0	10·0	10·0	10·0	10·0	10·0	10·0	10·0	10·0	10·0	10·0
Fishmeal Prime	10·0	10·0	10·0	10·0	10·0	10·0	10·0	10·0	10·0	10·0	10·0	10·0	10·0	10·0
Fishmeal by-product	10·0	10·0	10·0	10·0	10·0	10·0	10·0	10·0	10·0	10·0	10·0	10·0	10·0	10·0
Meat meal	10·0	10·0	10·0	10·0	10·0	10·0	10·0	10·0	10·0	10·0	10·0	10·0	10·0	10·0
Maize gluten	8·0	8·0	8·0	8·0	8·0	8·0	8·0	8·0	8·0	8·0	8·0	8·0	8·0	8·0
Blood meal	7·5	7·5	7·5	7·5	7·5	7·5	7·5	7·5	7·5	7·5	7·5	7·5	7·5	7·5
Diatomaceous earth	1·7	3·1	3·56	3·23	2·91	2·59	2·27	3·29	2·97	2·65	2·32	2	1·68	1·36
NaH_2_PO_4_	0·5	0·5	0·5	0·5	0·5	0·5	0·5	0·5	0·5	0·5	0·5	0·5	0·5	0·5
Vit/min premix	0·5	0·5	0·5	0·5	0·5	0·5	0·5	0·5	0·5	0·5	0·5	0·5	0·5	0·5
Rovimix Stay-C (35)	0·3	0·3	0·3	0·3	0·3	0·3	0·3	0·3	0·3	0·3	0·3	0·3	0·3	0·3
CC (70 %)	0·3	0·3	0·3	0·3	0·3	0·3	0·3	0·3	0·3	0·3	0·3	0·3	0·3	0·3
Met	0·2	0·2	0·2	0·2	0·2	0·2	0·2	1·11	1·11	1·11	1·11	1·11	1·11	1·11
Tau	0·0	0·32	0·64	0·97	1·29	1·61	1·93	0·0	0·32	0·64	0·97	1·29	1·61	1·93
Y_2_O_3_	0·1	0·1	0·1	0·1	0·1	0·1	0·1	0·1	0·1	0·1	0·1	0·1	0·1	0·1
Analysed composition (g/kg on a DM basis)														
Ash	117·0	115·8	119·3	118·6	117·4	116·2	115·4	116·1	116·6	117·2	114·9	113·5	111·0	110·0
Total lipid	144·5	149·0	158·4	154·1	152·8	151·5	157·7	178·3	161·3	155·8	160·6	139·9	144·6	154·3
Total protein	485·9	494·3	498·3	485·4	500·4	488·7	495·1	482·7	494·5	487·5	493·1	503·1	488·4	484·4
GE (MJ/kg)	21·9	22·0	22·2	22·2	22·1	21·9	22·9	21·8	22·0	22·1	22·3	22·2	22·2	22·7
Total Met	12·0	10·5	11·1	10·8	10·9	10·1	11·1	16·7	19·3	16·4	18·8	16·0	17·9	15·0
Total Cys	6·8	6·1	5·6	5·4	5·6	5·8	5·7	5·9	5·4	5·5	5·2	5·6	5·6	5·8
Total Tau	1·6	4·8	8·5	11·9	15·0	17·3	20·4	1·6	5·1	8·1	11·7	13·9	18·3	20·0

T + M, experimental diets from the TauMet study; CC, choline chloride; Met, methionine; Tau, taurine; Y_2_O_3_, yttrium; Cys, cysteine.

* Table 1 is adapted from Candebat *et al*.^(19)^

**Table 2.** List of measured responses and calculated ratios from the TauMet and MetCys study to assess yellowtail kingfish (*Seriola lalandi*) vital and health status

	Tissue	Study/diets	Measured response	Abbreviation
Liver colour	Whole liver	TauMet/T + M 1–14	L, a*, b* colour space	CIE Lab
			R, G, B colour space	RGB
			H, S, B colour space	HSB
Histomorphology and cytology	Liver	TauMet/T + M 1, 3, 4, 7, 8, 10, 11, 14	Bile duct wall thickness	BDW
			Fattiness	F
			Necrotic hepatocytes	NH
			Large nucleus	LN
			Marginated chromatin	MC
			Cytoplasm eosinophilia	E
Biochemistry	Blood plasma	TauMet/T + M 1–14	Cholesterol	
			TAG	
			Alkaline phosphatase	ALP
			Aspartate transaminase	AST
			Lactate dehydrogenase	LD
			Bicarbonate	
			Ca	
			Phosphate	
			Urea	
			Glucose	
			Lactate	
Macro- and histomorphology	Posterior intestine	TauMet/T + M 1, 4, 7, 8, 11, 14	Total fish weight	TW
			Viscera weight	VW
			Liver weight	LW
			Intraperitoneal fat weight	IPFW
			Posterior intestine circumference	PIC
			Total intestinal wall thickness	TIW
			Stratum compactum granulosum	S
			Muscularis interna thickness	MI
		MetCys/M + C 1, 2, 3, 4 Com I, E	Villus area	VA
			Lamina propria area	LPA
			Lamina epithelial area	LEA
			Total villus height	TVH
			Villus length	VL
			Total villi count	TVC
			Supranuclear vacuoles	SV
Histochemistry	Posterior intestine villi	TauMet/T + M 1, 4, 7, 8, 11, 14	Total goblet cell mucus	TGC
			AB+ goblet cell mucus	AB+
		MetCys/M + C 1, 2, 3, 4 Com I, E	PAS+ goblet cell mucus	PAS+
			AB + PAS+ goblet cell mucus	AB + PAS+
			Small PAS+ bullet-shaped bodies	S-PAS+

Throughout the present study, the second feeding trial by Candebat *et al.* ^(2)^ is referred to as the MetCys study, and the diets are annotated with M + C, whereas the commercial diet is annotated as Com. The objective of the MetCys study was to quantify the dietary Met requirements at Cys levels that met or exceeded industry practice^(2)^. Again, a factorial dose–response approach was applied, using five levels of Met, ranging from 7·9 to 25·2 g/kg, combined with either one of two levels of dietary Cys (5·6 or 13·9 g/kg) at one constant Tau level (7·0 (sem 0·03) g/kg; Table 3), resulting in ten experimental diets (M + C 1–10). Each of the ten experimental diets and a commercial diet were randomly assigned to three identical 200 l tanks, each stocked with twelve fish (initial body weight; 52·6 (sem 1·0) g/fish). After 8 weeks, the posterior intestines of six individual fish fed one of four diets, which contained the lowest or highest amount of Met and/or Cys, were collected (M + C 1:8·8 g Met/kg and 5·8 g Cys/kg, M + C 2:24·7 g Met/kg and 5·9 g Cys/kg, M + C3:7·9 g Met/kg and& 13·7 g Cys/kg, M + C 4:25·2 g Met/kg and 13·9 g Cys/kg). In addition, posterior intestines from six individual fish fed a commercial diet were collected for histological analysis (Table 2) at the start and end of the trial to provide baseline information on the relative effects on YTK health.

**Table 3.** Formulation and composition of the MetCys experimental diets

	Commercial diet	Low cysteine series		High cysteine series	
		M + C 1	M + C 2	M + C 3	M + C 4
Raw material composition (%DM basis)*					
Dehulled lupins	NA	19·91	19·91	19·91	19·91
Soya protein concentrate	NA	15·20	15·20	15·14	15·20
Fishmeal	NA	12·60	12·97	12·97	12·97
Gelatine	NA	14·33	12·73	13·45	11·86
Sodium caseinate	NA	9·78	10·10	9·78	10·10
Fish oil	NA	10·00	10·00	10·00	10·00
Blood meal	NA	5·00	5·00	5·00	5·00
Diatom. Earth	NA	5·17	5·13	4·40	4·54
Feather meal	NA	2·87	2·85	3·00	3·20
Maize starch	NA	2·50	1·33	2·60	1·33
NaH_2_PO_4_	NA	0·50	0·50	0·50	0·50
Vit-min premix	NA	0·50	0·50	0·50	0·50
CC (70 %)	NA	0·30	0·30	0·30	0·30
Rovimix Stay-C	NA	0·30	0·30	0·30	0·30
Lysine	NA	0·29	0·29	0·29	0·29
Met	NA	0·00	2·14	0·00	2·15
Cys	NA	0·00	0·00	1·11	1·10
Tau	NA	0·65	0·65	0·65	0·65
Y_2_O_3_	NA	0·10	0·10	0·10	0·10
Analysed composition (g/kg on a DM basis)					
Ash	40	107·3	108·2	100	104·3
Total lipid	140	135·8	135·2	130·2	138·1
Total protein	500	638·8	619·5	637·9	651·1
GE (MJ/kg)	NA	21·8	22·2	22·4	22·4
Total Met	12·6	8·8	24·7	7·9	25·2
Total Cys	6·5	5·8	5·9	13·7	13·9
Total Tau	7·5	7·0	6·9	7·0	6·9

M + C, experimental diets from the MetCys study; CC, choline chloride; Met, methionine; Tau, taurine; Y_2_O_3_, yttrium; Cys, cysteine.

* Table 3 is adapted from Candebat *et al*.^(2)^

Fish used in this study were the progeny of wild-caught YTK brood stock held at the Port Stephens Fisheries Institute hatchery. Juvenile YTK were fed a commercial floating pellet (crude protein 50 %, crude fat 14 % and crude fibre 4 %) and maintained at 15–19°C before experiment stocking. Throughout the feeding trials, fixed photoperiods of 12 light: 12 dark (TauMet study) and 11 light: 13 dark (MetCys study) were maintained, using dimmable, overhead LED lamps and simulating natural photoperiods of the season. Water quality parameters were monitored daily (mean) for the TauMet study: temperature (23·3 (se 0·6)°C), salinity (33·3 (se 4·6) %), dissolved oxygen (7·0 (se 1·0) mg/l), pH (8·3 (se 0·5) and TAN (0·7 (se 0·4) mg/l). For the MetCys investigation, the parameters were temperature (21·2 (se 0·6)°C), salinity (32·9 (se 3·2) %), dissolved oxygen (12·1 (se 3·0) mg/l), pH (7·4 (se 0·4)) and TAN (≤ 0·25 mg/l).

### Experimental diets and feeding regimens

All T + M and M + C diets were formulated to meet the protein and energy requirements of YTK^(33)^ and comprised of prime fishmeal, fisheries by-product meal, plant and terrestrial animal meals. The desired Met, Cys and Tau specifications for the T + M diets were achieved by using a blend of raw ingredients that were supplemented at different levels of crystalline dl-Met and Tau (Table 1), and for the M + C diets by using a blend of raw ingredients, crystalline dl-Met, l-Cys and Tau (Table 3). YTK were hand-fed to apparent satiation twice a day (at 09.00 and 15.00 hours) during weekdays and once per day at 09.00 hours on weekends for 45 d (TauMet study) and 54 d (MetCys study).

### YTK tissue collection: TauMet study

After the TauMet feeding trial, YTK were fasted for 24 h, and all fourteen YTK of each tank were individually measured for total length, whole body, fillet, viscera, liver and intraperitoneal fat weight. Six randomly selected fish per feeding treatment were euthanised by a spike to the brain, followed by immediate blood sampling. Approximately 1 ml of blood was collected from the caudal vein, using a 5 ml syringe and a 19 g × 38 mm gauge needle (Terumo). Collected blood was then rapidly transferred into lithium heparin-coated tubes (MiniCollect^®^Lithium Heparin) and centrifuged (LabCo^®^Mini Centrifuge) at 113 × 100 rpm for 14 min. Following centrifugation, the blood plasma was collected and pooled per experiment tank and frozen at −20°C until further biochemical analysis. The livers from the same six fish were removed and photographed under standardised light conditions for the digital liver surface colour analysis (see the ‘Ex vivo whole liver colour analysis’ section). Additionally, liver and posterior intestinal samples were collected from an additional six to eight fish fed diets T + M 1, 3, 4, 7, 8, 10, 11 and 14, after euthanising with the recommended dose of Aqui-S® (540 g/l isoeugenol; Aqui-S New Zealand Ltd) and transferred to 10 % buffered formalin for histological analysis.

### YTK tissue collection: MetCys and Com study

After the MetCys feeding trial, YTK were fasted for 24 h and euthanised as above. Four initial fish, subsampled at the commencement of the trial, and twelve YTK from each replicate tank at the conclusion of the trial were individually measured for total length, whole body, viscera and liver weight. The posterior intestines of the four initial fish and six of the twelve measured fish fed the diets M + C 1, 2, 3, 4 and Com (*n* 30) were sampled for posterior intestines and transferred in 10 % buffered formalin for histological analysis.

### Histology preparation and data collection

Formalin-fixed organs, collected from ninety-two YTK from the TauMet and MetCys studies, were routinely dehydrated using increasing concentrations of ethanol from 50 to 100 % (HistoCore Pearl Tissue Processor, Leica Microsystems Pty Ltd) and embedded in paraffin (HistoCore Arcadia C & H Embedding Center Leica Microsystems Pty Ltd) according to the standard histological procedure. Subsequently, samples were cut into ∼4 µM sections with a rotary microtome (∼4·0 mm thickness, CUT 4060 model, microTec GmbH) and mounted onto glass slides for histological staining.

Slides were stained with either haematoxylin–eosin, a combination of Alcian blue (AB, pH 2·5) and periodic acid-Schiff (PAS) or toluidine blue and followed a slightly adjusted staining protocols by the James Cook University Veterinary Pathology Department. The pH of the AB stain was 2·5 to stain most of the acid goblet cell mucus. Livers were stained with haematoxylin–eosin for semi-quantitative and quantitative morphometric and cytological evaluations. Posterior intestines were stained with AB-PAS for histomorphometric and histochemical evaluation of structures and the joint detection of neutral (PAS+; magenta), acid (AB+; blue) and mixed (AB + PAS+; purple) goblet cell mucus. Additionally, sections of posterior intestines were stained with toluidine blue; however, mast cells were not detected^(34)^. Prior to the data collection, histology slides of the posterior intestine and liver were scanned at 40× magnification using an automated slide-scanning system (Aperio LV1 IVD, Leica Microsystems Pty Ltd). Aperio ImageScope software (Leica Biosystems) was used to visualise and measure the stained organs.

**Fig. 1.**
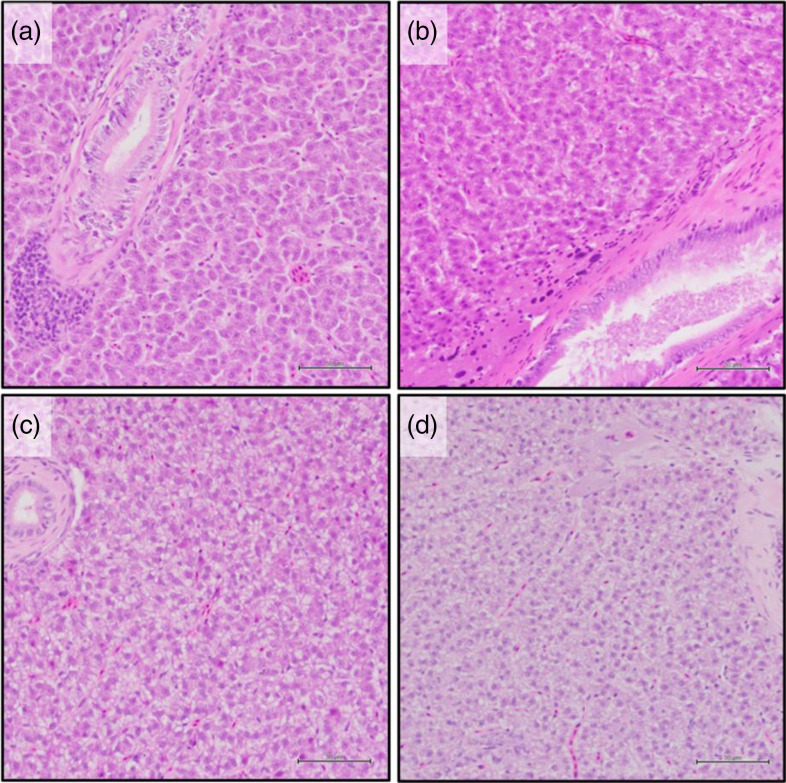
Semi-quantitative scoring of yellowtail kingfish (*Seriola lalandi*) liver collected from the TauMet study for the presence of lipid vacuoles, indicating fattiness/steatosis. The four levels are (a) 0 – normal; (b) 1 – mild; (c) 2 – moderate; (d) 3 – severe (Haematoxylin–eosin stain, scale bar = 50 µm).

**Fig. 2.**
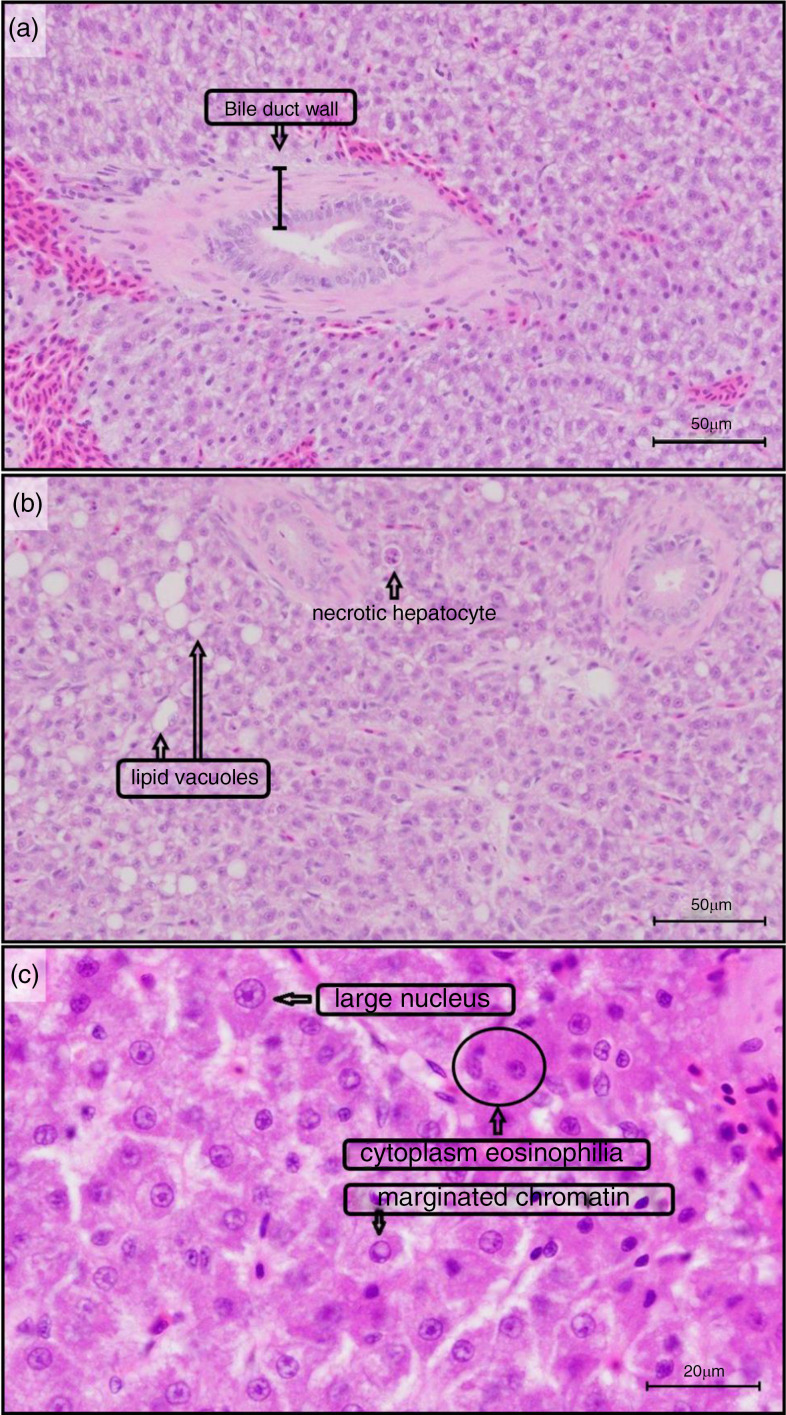
Histological features measured in *yellowtail kingfish (Seriola lalandi)* liver (TauMet study) fed one of seven dietary tauriness and one of two methionine levels. YTK liver was measured and quantified for (a) bile duct wall thickness (fibrous wall + epithelium), (b) necrotic hepatocytes, lipid vacuoles (semi-quantitative, see Fig. 1), (c) large nucleus of hepatocytes, cytoplasm eosinophilia in hepatocytes and marginated chromatin. (Haematoxylin–eosin stain, scale bar = 50 µm (a, b) and 20 µm (c)).

YTK livers collected from the TauMet study were scored for fattiness using a semi-quantitative scale (Table 2; Fig. 1). Liver fattiness was ranked from one to four by the abundance of lipid vacuoles in eighteen areas of interest (0·094 mm^2^/area) per liver section (Fig. 1). The quantitative system included measurements and counts on bile duct wall (BDW) thickness, including the fibrous wall and cholangiocytes, of which the five thickest bile ducts were selected for subsequent statistical analysis (Fig. 2(a)), necrotic hepatocytes, (Fig. 2(b)), large nucleus, cytoplasm eosinophilia ® and marginated chromatin in hepatocytes in three 400× fields (0·094 mm^2^/area), respectively (Fig. 2(c)).

**Fig. 3.**
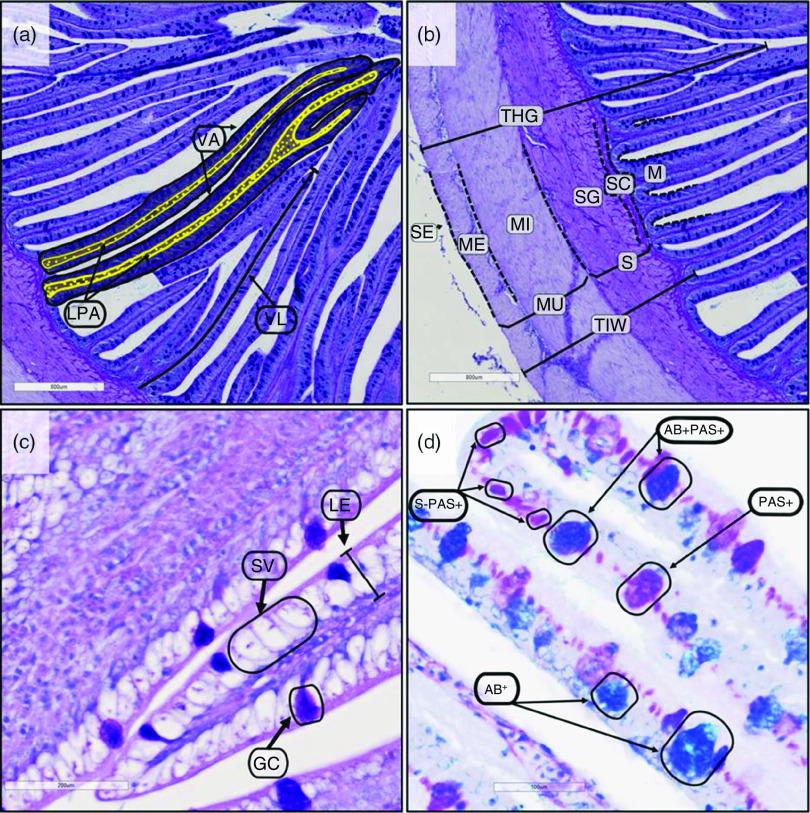
Posterior intestinal structures of juvenile yellowtail kingfish (Seriola lalandi). A (M) following descriptions indicate that structure was measured and statistically analysed. (a): VA, villus area; LPA, lamina propria area (yellow area); VL, villus length; (b): TVH, total villus height (M); MU, muscularis; ME, muscularis externa; MI, muscularis interna (M); SC, stratum compactum; SG, stratum granulosum; S, submucosa (M); M, mucosa; TIW, total intestinal wall thickness (M); (c): LE, lamina epithelial; SV, supranuclear vacuoles (M); GC, goblet cells (M); (d): AB+, mucus that stained blue with Alcian blue (blue) (M); PAS+, mucus that stained with periodic acid-Schiff’s (magenta) (M); AB + PAS+, Alcian blue – periodic acid-Schiff’s positive stain mucus (purple) (M); S-PAS+, small periodic acid-Schiff’s dense bullet-shaped bodies (magenta and 18·6 (se 0·7) µm) (M). (AB-PAS stain, scale bar = 800 µm (a–b), 200 µm (c), 100 µm (d)).

Histological scans of the YTK posterior intestine collected from the TauMet and MetCys studies were quantitatively measured, including measurements of the length, thickness, area and counting structures within the organs (Fig. 3). Measured lengths included total intestinal wall (TIW) thickness, muscularis interna (MI) thickness, stratum compactum + granulosum thickness (S) of thirty-two different areas along the intestine per individual, total villus height (Fig. 3(b)) and villus length (VL) of twelve villi per individual (Fig. 3(a)). Measured areas included villus area (VA) and lamina propria area (LPA) of twelve villi per individual (Fig. 3(a)). Measured circumferences included the posterior intestine circumference (PIC) per individual. Quantified features of villi included total villi count per individual and villus tip count of twelve villi per individual. Histochemical features that were assessed included neutral, acidic and mixed goblet cell mucus, small PAS + dense bullet-shaped bodies (S-PAS+; magenta) and supranuclear vacuoles of twelve villi per individual (Fig. 3(d)). Prior to counting, a brightness/contrast filter was applied to digitised slides, enhancing the distinct colourations of acidic, neutral, mixed goblet cell mucus and supranuclear vacuoles.

### Calculations and statistical analysis

Statistical analyses were performed using IBM SPSS statistics, the R software environment for statistical computing (2.13) and R Studio v.4.0., using the R packages car, carData, ggplot2, ggpubr, multcompView, plyr and PMCMRplus. All dependent variables from the TauMet and MetCys study were subjected to two-way ANOVA to examine the effect of seven dietary Tau levels at each of two dietary Met levels (TauMet study) or five dietary Met levels at each of two dietary Cys levels (MetCys study). Prior to statistical analysis, dependent variables were validated for assumptions of (1) normality via Shapiro–Wilk test and (2) homogeneity of variance via Levene’s test. If assumptions were not met, dependent variables were log, sqrt, ln and inverse transformed. Further, dependent variables were assessed for the assumptions of (3) linearity with covariates including body weight and VA and (4) homogeneity of regression slopes between treatments. If assumptions 3 and 4 were met, dependent variables were subjected to two-way ANCOVA to control for the influence of the covariate (final body weight or VA). All dietary treatment means within the feeding trial were compared via Tukey honestly significant difference (HSD) post hoc test in the event of a significant interaction. Similarly, in the event of no significant interaction but significant main factor effects, the respective factor level means were compared using Tukey HSD post hoc test^(35)^.

An abbreviation list on the measured responses can be found in Table 2. The LPA and lamina epithelial area indicated a strong and significant relationship with VA and were therefore controlled through a two-way ANCOVA to examine the effects of dietary Met, Cys and Tau. Further, the density of AB + goblet cell mucus, PAS+ goblet cell mucus, AB + PAS+ goblet cell mucus, total goblet cell mucus, small PAS+ dense bullet-shaped bodies and supranuclear vacuoles within the respective VA was calculated and statistically analysed (LPA/VA; LEA/VA; AB+/VA; PAS+/VA; AB + PAS+/VA; TGC/VA; S-PAS+/VA; SV/VA). The semi-quantitative liver data were subject to a non-parametric Kruskal–Wallis test. Effects were considered significant at *P* < 0·05. Pearson’s correlation coefficients between liver colour values and TDW thickness, fattiness, plasma cholesterol and TAG were calculated using Excel.

### Ex vivo whole liver colour analysis

Image acquisition methods were adapted from Trampel *et al.* ^(36)^. Before image acquisition, livers were visually assessed for whole or partial green discolouration. Images of eighty-four ex vivo YTK liver (TauMet study) were taken under standardised light and object orientation conditions. All images were captured using a digital SLR camera (Sony ILCE-6300) with a 16 mm lens and fixed settings of ISO-250, 1/13 s shutter speed, a focal length of 20 mm and aperture ƒ/6·3. Livers were carefully patted dry with paper towels before being placed individually on an 18 % grey card background (8”×10”, Delta 1) inside a 290 × 450 mm closed photo box. The camera was placed in a slot at the top of the photo box, creating a perpendicular distance of 250 mm between the camera and liver samples. The photo box was made of white polypropylene, and the walls served as a diffuser to minimise the glare of lighting. The container was illuminated with 2 × 11 W white LED lights (Mirabella) placed 20 cm outside the longitudinal walls of the photo box. Additionally, the images were taken in a dark room, away from daylight, to standardise the lighting conditions. The colour assessment methodology applied to YTK liver was adapted from Weller & Westneat^(37)^ and van Belleghem *et al*.^(38)^.

Average liver colour, liver colour composition and colour distance of individual YTK liver samples (TauMet study) were assessed. Image backgrounds, blood vessels on the liver and glare were removed to reduce bias from these artifacts. Liver images were compared via RStudio v.4.0. (colour distance package) by distinguishing colour intensity and relative proportion of red, green, blue (RGB) metrics, followed by colour distance calculations to quantify the degree of similarity or dissimilarity between two images^(37)^.

Colour distance values were plotted using a principal coordinate analysis (PCoA) graph, visualising dissimilarity by distance and similarity by clustering. This was achieved via RStudio v.4.0., using the R packages ggrepel, devtools, colordistance, ComplexHeatmap, splitstackshape, readxl, ggpubr and cowplot.

The colour composition of each liver was assessed using ImageJ software with the 3D Color Inspector/colour histogram plugin^(39)^. Three areas of each liver were selected (46·441 (se 897) pixels/area and 51·2 (se 0·8) colours/area) to provide information on RGB and distribution. The average colour of ex vivo livers was assessed via Adobe Photoshop 2021 by applying a blur filter on the extracted liver areas and calculating the average value in CIE Lab (luminance, red–green, blue–yellow channels), RGB (red, green and blue channels) and HSB (hue, saturation and brightness)^(40)^. The average colour was then sampled with the Photoshop eyedropper tool.

## Results

### TauMet study: liver histology

**Fig. 4.**
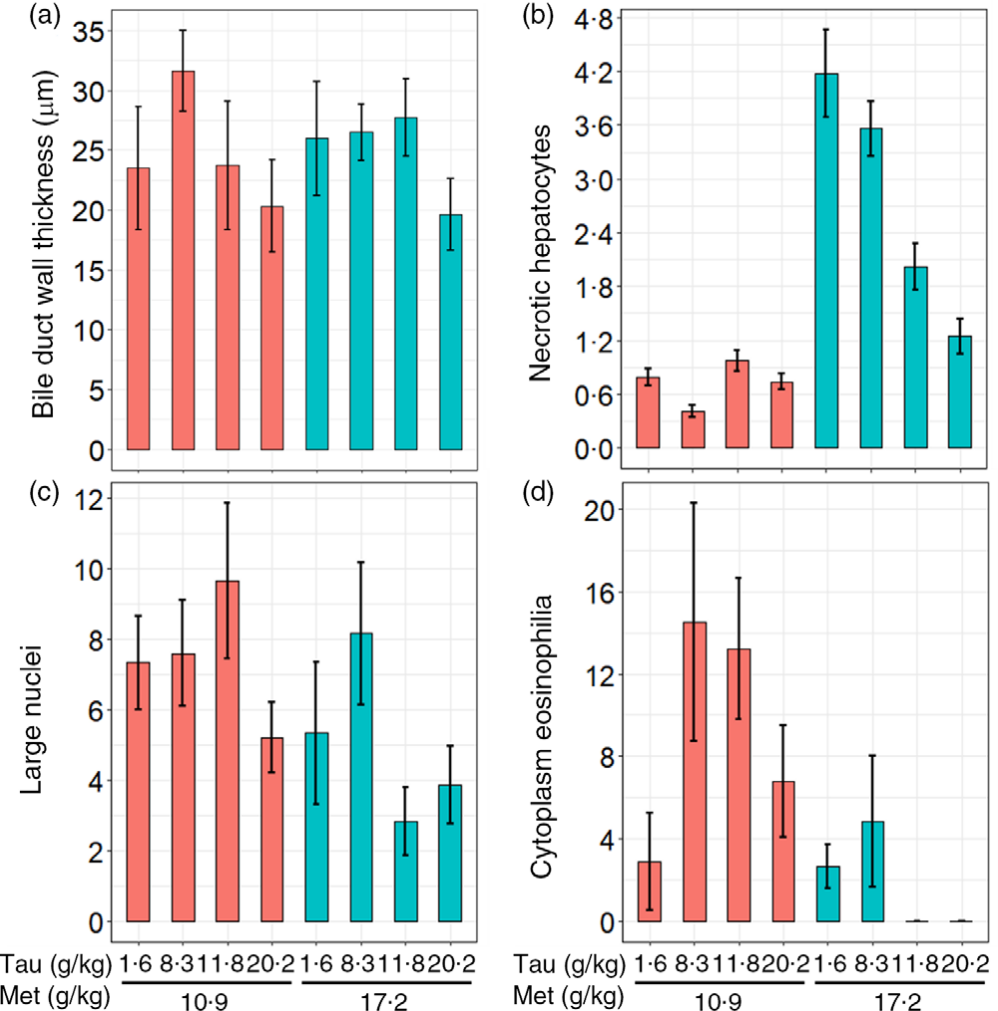
Liver histology of juvenile yellowtail kingfish (Seriola lalandi) (TauMet study), fed one of six taurines and one of two methionine levels. (a) Bile duct wall thickness (µm), (b) number of necrotic cells per 0·094 mm^2^, (c) count of large nuclei per 0·094 mm^2^, (d) count of cytoplasm eosinophilia in hepatocytes per 0·094 mm^2^. Data expressed as mean values with their standard errors.

Overall, the histological appearance of the livers was normal relative to that of farmed fish, with fatty livers and irregularities being minor in all treatments. Dietary Tau and Met did not statistically affect the score of the semi-quantitative fattiness measurement; however, quantitative measurements of the BDW thickness, necrotic hepatocytes, cytoplasmic eosinophilia and large nuclei in hepatocytes were all modulated by diet (Table 2). YTK fed the highest levels of dietary Tau (T + M 7 and 14) had significantly thinner BDW (Fig. 4). Further, an increase of necrotic cells with dietary Met at 17·2 g/kg was observed. Yet, an incremental increase of Tau at 17·2 g Met/kg reduced the appearance of necrotic hepatocytes. YTK fed diets containing dietary Met at current industry practice (11 g Met/kg diet) had more and larger hepatocyte nuclei (7·45 (se 0·4) per 94 µm^2^) than YTK fed Met levels above current industry practice (5·05 (se 1·5) per 94 µm^2^). Cytoplasm eosinophilia in hepatocytes was most pronounced in YTK fed diet T + M 3 at 14·5 (se 1·3) per 94 µm^2^ and was on average higher in YTK fed dietary Met at 10·9 g/kg; however, dietary Met and Tau did interact. Various hepatocytes also exhibited marginated chromatin, which was most pronounced in YTK fed diet T + M 4 (13·6 (se 1·6) per 94 µm^2^); however, there were no significant differences among treatments.

### TauMet study: plasma biochemistry

**Fig. 5.**
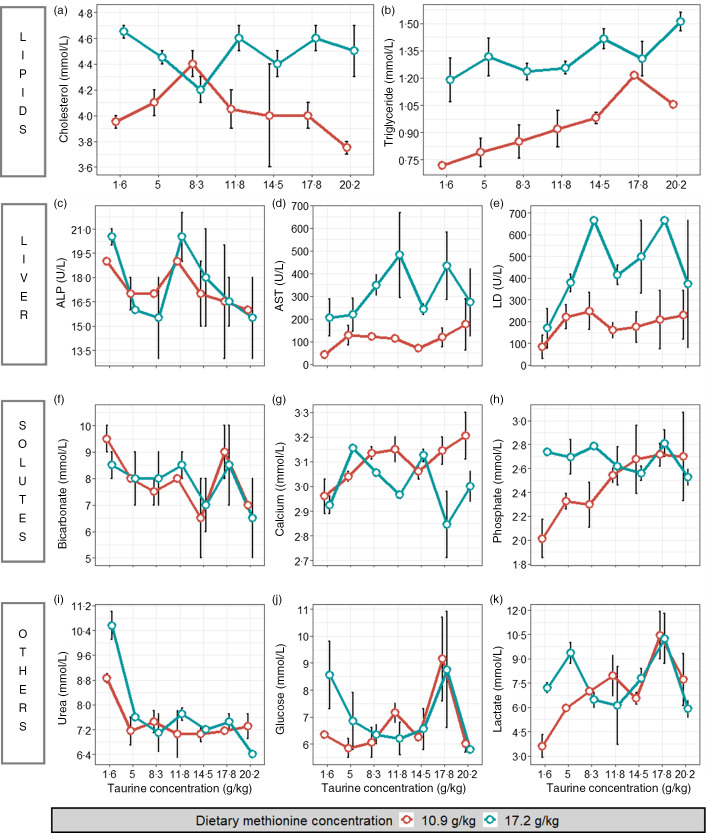
Plasma chemistry of juvenile yellowtail kingfish (Seriola lalandi) fed diets containing one of six different taurine levels and one of two methionine levels. Panels (a–b) show results on lipoproteins; panels (c–d) show results on liver function tests. ALP, alkaline phosphatase (c); AST, aspartate transaminase (d); LD, lactate dehydrogenase (e); panels (f–h) show results on solutes that form electrolytes and panels (i–k) show results on other plasma chemistry results. Red lines are the low met series (10·9 g Met/kg diet), and blue lines are the high methionine series (17·2 g Met/kg diet) at varying levels of taurine. The range bars indicate the two collected values.

Plasma from YTK in this study had in comparison a mild elevation to depression of plasma biochemical parameters (Fig. 5). Further, trends of blood biochemistry indicated Tau- and Met-dependent changes. Cholesterol and TAG contents in plasma did not exceed 4·7 and 1·6 mmol/l, respectively. However, lipid contents were generally higher in YTK fed 17·2 g Met/kg. The enzymatic activity of alkaline phosphatase, aspartate transaminase (AST) and lactate dehydrogenase (LD) appeared to be either responsive to dietary Tau or Met. YTK fed diets containing 17·2 g Met/kg exhibited higher AST and LD activity than YTK fed diets containing Met at current industry practice. Solute contents of bicarbonate, Ca and phosphate indicated responsiveness to changes in dietary Tau concentrations, where bicarbonate decreased from 10 to 5 mmol/l, and phosphate and Ca increased from 1·85 to 3·07 mmol/l and 2·7 to 3·3 mmol/l, respectively, with incremental increases in dietary Tau.

### TauMet study: liver surface colour

**Fig. 6.**
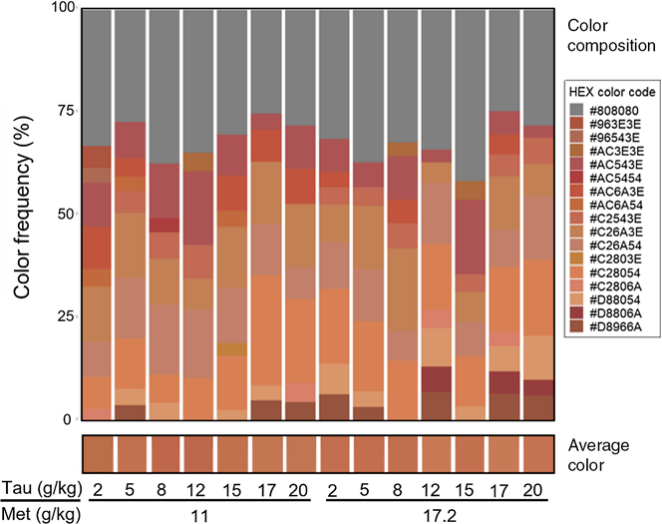
(a) Distribution of juvenile yellowtail kingfish (Seriola lalandi) liver surface colours across dietary treatments from the TauMet study. Grey sequences are the collection of colours that were each ≤ 3 % present in the total liver surface colour composition (*n* 6) within a diet. Coloured sequences are tinted in the respective hex colour code and represented ≥ 3 % of the total liver colour composition (*n* 6). (b) Average RGB liver surface colour of each dietary treatment from the TauMet study. Average RGB values were converted to a single-colour square that represents the average liver colour of liver tissue (*n* 6).

The visual and subjective liver surface colour assessment indicated no green liver syndrome in YTK. On subjective and macroscopic assessment, liver surface colours were mostly red–orange–yellow–peach. On an objective, digital assessment liver surface colours were close to the hex colour ‘copper-red; #CB6D51’. The digital assessment of the liver surface colour demonstrated that the changes in green–red (a*), r®® and brightness (B) values were dependent on dietary Tau and Met (Table 4). The a* value of liver surface colour was at the highest (the reddest) when juvenile YTK were fed diet T + M 3 (8·5 g Tau/kg and 10·9 g Met/kg), at which the dietary Tau level was closest in meeting the Tau requirement at 10·9 g Met/kg^(19)^. Feeding dietary Met at 17·2 g/kg resulted in significantly higher red (R) and blue (B) values of liver surface colour compared with feeding Met levels at 10·9 Met/kg (Fig. 6; Table 4).

**Table 4.** Juvenile yellowtail kingfish (*Seriola lalandi*) liver surface colour components from the TauMet study (*n* 6) expressed in CIE, RGB and HSB colour model. T + M 11 is closest to the average TSAA (Met+Cys) requirement of 24·5 g/kg diet and the methionine-dependent taurine requirement of 7·7 g/kg diet^(2,19)^ (Mean values with their standard errors)

	Experimental diet																												*P*		
	T + M 1		T + M 2		T + M 3		T + M 4		T + M 5		T + M 6		T + M 7		T + M 8		T + M 9		T + M 10		T + M 11		T + M 12		T + M 13		T + M 14		T	M	T * M
	Mean	se	Mean	se	Mean	se	Mean	se	Mean	se	Mean	se	Mean	se	Mean	se	Mean	se	Mean	se	Mean	se	Mean	se	Mean	se	Mean	se			
Dietary taurine, methionine and cysteine contents (g/kg diet)																															
T	1·6		4·8		8·5		11·9		15·0		17·3		20·4		1·6		5·1		8·1		11·7		13·9		18·3		20·0		–	–	–
M	12·0		10·5		11·1		10·8		10·9		10·1		11·1		16·7		19·3		16·4		18·8		16·0		17·9		15·0		–	–	–
C	6·8		6·1		5·6		5·4		5·6		5·8		5·7		5·9		5·4		5·5		5·2		5·6		5·6		5·8		–	–	–
CIE Lab (lightness, red–green, blue–yellow) colour model																															
L	55·2	1·2	56·4	0·9	53·8	0·9	53·6	1·6	56·6	1·3	58·0	1·5	57·6	1·4	55·8	1·5	56·0	1·5	56·0	2·2	59·4	1·8	56·5	1·4	59·4	1·8	57·2	1·1	NS	NS	NS
a	28·8	0·7^a,b^	31·0	1·2^a,b^	34·2	0·7^a^	34·0	1·5^a,b^	30·2	1·0^a,b^	28·2	1·2^b^	28·4	1·7^a,b^	31·2	1·3^a,b^	32·0	1·3^a,b^	31·2	2·0^a^	29·4	2·1^a,b^	31·5	1·6^a,b^	28·6	1·2^b^	31·0	1·7^a,b^	*	NS	NS
b	33·6	1·4	32·8	0·6	31·6	1·6	30·8	1·1	33·8	1·2	33·4	0·2	32·4	0·9	32·0	0·8	33·0	0·5	33·0	1·5	33·4	1·4	33·2	1·5	33·2	1·0	33·2	1·2	NS	NS	NS
RGB (red, green, blue) colour model																															
R	187·2	3·1^a^	194·6	1·2^a^	190·8	2·0^a^	189·8	2·5^a^	194·2	2·2^a^	195·4	2·9^a^	194·8	2·5^a^	193·8	2·8^b^	195·0	3·0^b^	193·6	4·8^b^	201·6	2·6^b^	194·8	3·0^b^	200·2	4·4^b^	196·8	1·6^b^	NS	**	NS
G	110·8	3·4	112·6	3·1	103·2	3·0	103·0	5·3	113·6	3·9	118·8	4·9	117·6	5·2	111·2	4·8	110·8	4·7	111·2	7·3	121·4	6·4	112·0	5·1	122·6	5·2	114·6	4·0	NS	NS	NS
B	74·2	3·4	79·6	2·0	75·4	1·6	77·0	3·0	78·2	4·0	82·0	3·0	82·8	2·9	80·2	2·5	78·4	2·7	78·2	5·0	86·0	4·4	78·8	3·4	87·0	5·1	81·0	2·7	NS	NS	NS
HSB (hue, saturation, brightness) colour model																															
H	19·4	1·4	17·4	1·1	14·4	1·7	13·8	1·8	18·4	0·9	19·6	0·9	18·4	1·5	16·4	1·3	16·6	1·1	17·0	2·0	18·2	1·8	17·0	1·8	18·8	1·2	17·6	1·6	NS	NS	NS
S	60·6	1·4	59·0	0·9	60·6	1·1	59·6	1·2	59·2	1·5	58·0	0·9	57·6	1·1	58·8	0·9	60·0	0·8	59·8	1·9	57·2	1·8	59·8	1·4	57·2	1·8	58·8	1·1	NS	NS	NS
B	73·4	1·2^a^	76·2	0·5^a^	74·8	0·7^a^	74·4	1·0^a^	76·2	0·7^a^	76·6	1·1^a^	76·8	1·0^a^	76·4	1·0^b^	76·6	1·1^b^	76·2	1·8^b^	79·0	1·0^b^	76·2	1·1^b^	78·6	1·8^b^	77·2	0·6^b^	NS	**	NS

T, taurine; M, methionine; C, cysteine.

Levels of significance were assessed via two-way ANOVA and are with respect to

* *P* < 0·05,

** *P* < 0·01

^a,b,c^Data with the same superscript letter within rows are statistically similar (*P* < 0·05).

The average colour of the liver surface, as indicated by the hex code, of each T + M diet was distinct (Fig. 6). However, the liver surface colour is composed of main colours, which recurred in all dietary treatments, but at varying proportions (Fig. 6).

**Fig. 7.**
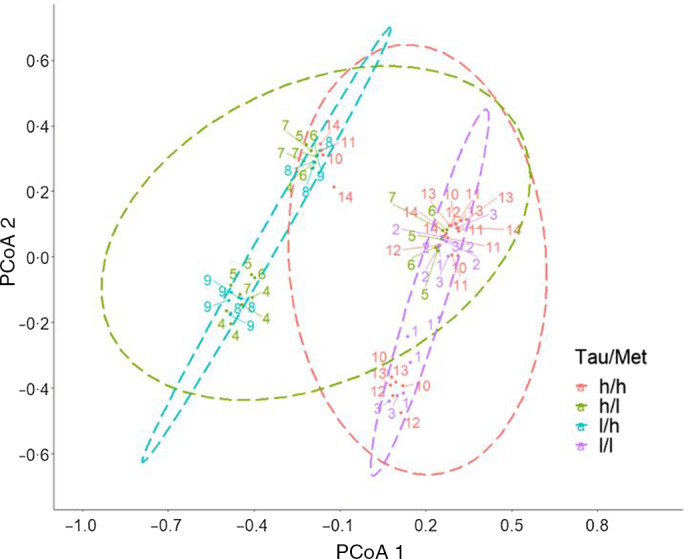
PCoA using the colour distance method and clustered by similarity of individual juvenile yellowtail kingfish (*Seriola lalandi*) liver surface colours from the TauMet study. A dot represents an individual liver, and numbers correspond to the respective diet. Ellipses (dashed lines) indicate distribution at 95 % confidence level of Tau/Met levels at high/high (h/h in red), high/low (h/l in green), low/high (l/h in blue) and low/low (l/l in purple). PCoA, principal coordinate analysis.

The colour distance between the individual liver surface colours ranged from 0 to 1 in a heatmap, and the conversion into a PCoA distribution indicates the colour distance between the T + M diets (Fig. 7), identifying a division into four colour clusters. Livers of juvenile YTK fed the T + M diets that were both relatively low in dietary Tau were separated along PCoA 1 by relatively high dietary Met (Fig. 7; l/h in blue) and low Met (Fig. 7; l/l in purple) and did not fall within each other’s 95 % confidence ellipse. However, individuals fed relatively high Tau and Met (Fig. 7; h/h in red) and relatively high in Tau and low in Met (Fig. 7; l/h in blue) are separated along the negative PCoA 2 axis and then mix along the positive PCoA2 axis, as shown by the overlap of the 95 % confidence ellipse.

### TauMet study: relationship of liver surface colour with biochemistry and histology

Weak to strong relationships between liver surface colour and liver histology and plasma biochemistry were found (Table 5). The BDW thickness correlated positively with the green–red channel (a*, CIE) and negatively with the blue–yellow (b*, CIE Lab) and hue channel (H, HSB) of the measured liver surface colour. The semi-quantitative fattiness scores had a positive correlation with blue–yellow (b*; CIE Lab) and hue (H; HSB). Blood plasma cholesterol correlated positively with red and blue (R, B; RGB) and the brightness (B; HSB) of liver surface colour. Blood plasma TAG contents correlated positively with the perceptual lightness (L; CIE Lab), red and blue (R, B; RGB) and brightness (B; HSB) of the liver surface colour.

**Table 5.** Pearson correlation coefficients for liver colouration values and bile duct wall thickness, liver fattiness, blood plasma cholesterol and TAG contents of juvenile yellowtail kingfish (*Seriola lalandi*)

	Bile duct wall thickness	Hepatic fattiness	Cholesterol (mmol/l)	TAG (mmol/l)
CIE Lab colour model (lightness, green–red, blue–yellow)				
L	–0·12	0·48	0·38	0·54
a*	0·57	–0·45	0·14	–0·19
b*	–0·41	0·89	–0·14	0·24
RGB (red, green, blue) colour model				
R	0·14	0·34	0·57	0·60
G	–0·23	0·50	0·28	0·49
B	0·05	0·16	0·56	0·55
HSB (hue, saturation, brightness) colour model				
H	–0·56	0·67	–0·17	0·20
S	0·02	0·00	–0·45	–0·42
B	0·15	0·35	0·55	0·60

### TauMet study: posterior intestine

The posterior intestinal wall of YTK (250–327 g) ranged from 1 to 1·5 mm thick (Table 6). The intestinal wall was composed of a thin outer serosa layer, a muscularis layer consisting of a muscularis externa and interna (0·4–0·7 mm), a submucosa (0·3–0·4 mm) consisting of a stratum granulosum (2 longitudinal × 2 circular layers), stratum compactum and a mucosa consisting of a lamina propria (149–355 mm^2^) and lamina epithelial. Each 4 µm thick section of the intestine had between 68 and 137 villi (total villi count) that ranged from 3·9 to 5·6 mm in length (VL). The villi were complex, branching into several villus tips.

**Table 6.** Macromorphometric and histomorphometric features of juvenile yellowtail kingfish (*Seriola lalandi*) posterior intestine, fed one of six different taurine-methionine levels from the TauMet feeding trial. T + M 11 is closest to the average TSAA (Met+Cys) requirement of 24·5 g/kg diet and the methionine-dependent taurine requirement of 7·7 g/kg diet in YTK^(93)^ (Mean values with their standard errors)

	Diets												Model	*R* ^2^	*P*		
	T + M 1		T + M 4		T + M 7		T + M 8		T + M 11		T + M 14				T	M	T * M
	Mean	se	Mean	se	Mean	se	Mean	se	Mean	se	Mean	se					
Dietary taurine and methionine contents (g/kg diet)																	
T^1^	1·6		11·9		20·4		1·6		11·7		20·0		–	–	–	–	–
M	12·0		10·8		11·1		16·7		18·8		15·0		–	–	–	–	–
C	6·8		5·4		5·7		5·9		5·2		5·8		–	–	–	–	–
Macromorphometrics (g, unless otherwise stated)																	
BW	143·3	1·7^a^	176·8	3·1^a,b^	173·4	4·8^a^	198·7	4·8^a,b^	231·8	3·9^b^	230·7	3·6^b^	M1^2^	–	*	***	NS
VW	9·1	0·1^a^	10·9	0·2^a,b^	10·4	0·2^a^	13·2	0·3^b^	13·3	0·2^b^	13·2	0·2^b^	M1	–	NS	***	NS
VSI (%)	6·4	0·05^a^	6·2	0·03^b^	6·1	0·05^b^	6·6	0·03^a^	5·8	0·03^b^	5·8	0·04^b^	M1	–	***	NS	NS
LW	1·2	0·2^a^	1·5	0^a,b,c^	1·4	0·0^a,b^	1·7	0·0^a,b,c^	1·9	0·0^b,c^	2·0	0·0^b,c^	M1	–	*	***	NS
HSI (%)	0·82	0·01	0·84	0·01	0·83	0·01	0·83	0·01	0·83	0·01	0·87	0·01	M1	–	NS	NS	NS
IPFW	0·6	0·0^a^	0·8	0·0^a^	0·7	0·0^a^	1·2	0·1^b^	1·3	0·1^b^	1·4	0·0^b^	M1	–	NS	***	NS
IPFI (%)	0·45	0·01^a^	0·44	0·02^a^	0·38	0·01^a^	0·59	0·02^b^	0·53	0·02^b^	0·59	0·02^b^	M1	–	NS	**	NS
Histomorphometry of the posterior intestine (µm, unless otherwise stated)																	
PIC	45 655	441^a^	39 599	984^a^	40 820	870^a^	46 387	553^b^	47 538	675^b^	45 662	474^b^	M1	0·04	NS	*	NS
TVH	4930	107^a,b^	4598	85^a^	5293	127^b,c^	5637	185^c^	5644	127^c^	5750	124^c^	M1	0·06	**	***	NS
VL	4493	177^a,b^	3946	152^a^	5182	220^b,c^	5626	287^c^	5057	187^b,c^	5261	207^b,c^	M1	0·01	**	***	*
TIW	1064	14^a^	1155	17^a^	1289	27^a^	1445	26^b^	1271	26^b^	1491	27^b^	M1	0·21	NS	**	NS
MI	443	7^a^	528	16^ab^	531	7^a,b^	624	9^b,c^	564	9^a,b^	735	6^c^	M1	0·19	*	**	*
S	324	2	367	10	393	10	401	9	347	3	420	8	M1	0·14	NS	NS	NS
TV	125	1^a^	114	2^a,b^	101	2^b^	118	2^a^	110	1^a,b^	107	2^b^	M1	0·03	*	NS	NS
VA (mm^2^)	1252	65^a^	975	49^a,b^	1510	86^b,c^	1608	77^c^	1706	88^c^	1821	112^c^	M1	0·05	***	***	*
LEA (mm^2^)	1044	53^a^	826	41^a^	1260	72^a^	1311	66^a,b^	1416	74^a^	1466	90^b^	M2^3^	0·99	NS	NS	***
LPA (mm^2^)	209	13^a,d^	149	9^a,b,c,d^	249	16^a,d^	297	15^b,c,d^	290	16^a,d^	355	24^bc^	M2^3^	0·80	***	***	NS

T, taurine; M, methionine; C, cysteine; BW, total fish weight; VW, viscera weight; VSI, viscerosomatic index; LW, liver weight; HSI, hepatosomatic index; IPFW, intraperitoneal fat weight; IPFI, intraperitoneal fat index; PIC, posterior intestine circumference; TVH, total villus height; VL, villus length; TIW, total intestinal wall thickness; MI, muscularis interna thickness; S, submucosa thickness; TV, total villi count; VA, villus area; LPA, lamina propria area, LEA, lamina epithelial area.

The significant effects were determined by two-way ANOVA (M1) or ANCOVA (M2) and levels of significance are with respect to

* *P* < 0·05,

** *P* < 0·01 and

*** *P* < 0·001.

^a,b,c^Data with the same superscript letter within rows are statistically similar (*P* > 0·05).

LPA and LEA were controlled via two-way ANCOVA for villus area prior to the two-way ANOVA; however, reported values are uncontrolled and expressed as mean values with their standard errors.

**Fig. 8.**
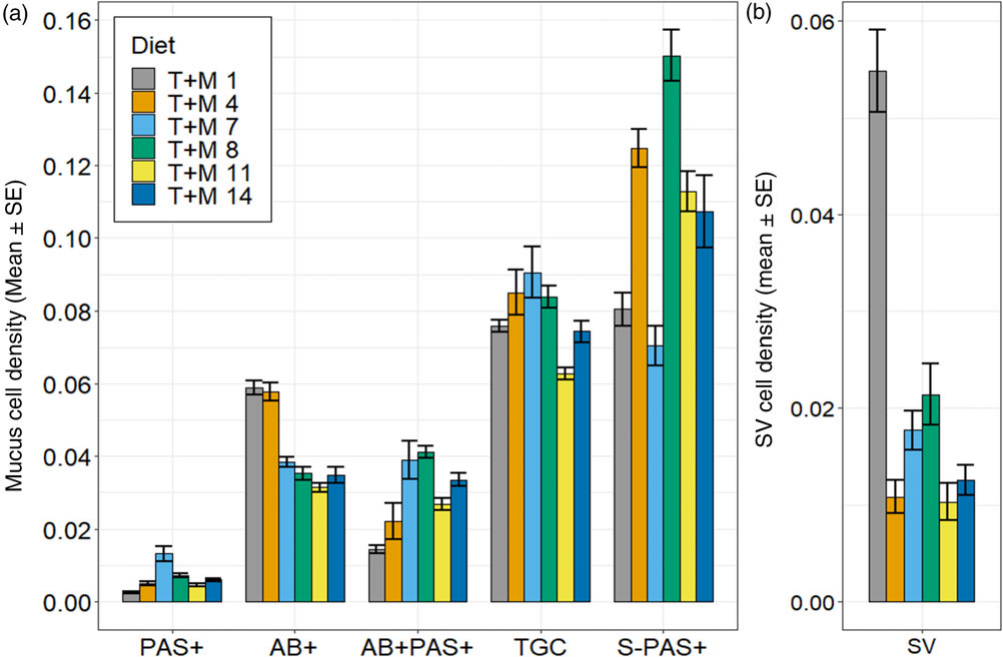
Barplots on the histochemical analysis per villus area of the PI of juvenile yellowtail kingfish (Seriola lalandi) fed one of six taurine-methionine combinations (TauMet). (a) Neutral goblet cell mucus (PAS+ per villus area), acid goblet cell mucus (AB+ per villus area), or mixed goblet cell mucus (AB + PAS+ per villus area), total goblet cell mucus (TGC per villus area) and bullet-shaped PAS+ mucus (S-PAS+ per villus area). (b) Supranuclear vacuole density (SV per villus area). Error bars indicate standard error.

Histomorphometric measurements were tested for possible effects of body weight and VA before being subjected to two-way ANOVA. However, relationships violated linearity and were not statistically significant; thus, data were not controlled. Exceptions were the measured lamina epithelial and LPA, which strongly correlated with the VA and met assumptions to adjust means for VA via two-way ANCOVA. YTK fed the diets T + M 8, 11 and 14 exhibited increased PIC, total villus height, VL, VA, lamina epithelial surface, TIW and MI thickness, whereas YTK fed less dietary Tau and Met (e.g. diet T + M 1) had less intestinal surface area, for example, PIC, total villus height, VL, TIW and MI. However, YTK fed less dietary Tau had more villi, in which number decreased when YTK were fed more dietary Tau. The intestinal mucosa did not exhibit necrosis or acute inflammation. Nevertheless, the VA-controlled LPA were responsive to dietary Met and Tau. Overall, YTK fed dietary Met at 17·2 g/kg exhibited greater LPA, which may indicate increased lymphocytes, macrophages and eosinophilic granule cell accumulations. The histochemical analysis of the posterior intestine revealed three types of mucus from goblet cells that stained: acidic, neutral and mixed (Fig. 8(d)). Goblet cell mucus predominately stained acidic AB+ (53–73 per villus) or mixed (18–60 per villus), whereas only a few mucus cells stained neutral (TauMet study: 3–14 per villus; MetCys study: 4–16 per villus; Fig. 8). The posterior intestines revealed a range of Tau and Met induced histochemical changes (Fig. 8).

The total goblet cell mucus production was most pronounced in fish fed the T + M 7 diet, exhibiting the greatest total goblet cell density (TGC/VA; Fig. 8; Table 7). Dietary Tau and Met induced significant changes in the composition of goblet cell mucus types, where increasing levels of dietary Tau and Met decreased the density of acidic (AB+) goblet cell mucus. Increasing levels of dietary Tau at 10·9 g Met/kg increased mixed and neutral goblet cell mucus; however, at Met levels above current industry practice, increasing dietary Tau led to a decrease in mixed and neutral goblet cell mucus (Fig. 8).

**Table 7.** Histochemical analysis of intestinal mucus and supranuclear vacuoles in juvenile yellowtail kingfish (*Seriola lalandi*) fed one of six taurine-methionine levels (TauMet). T + M 11 is closest to the average TSAA (Met+Cys) requirement of 24·5 g/kg diet and the dependent taurine requirement of 7·7 g/kg diet in YTK^(93)^ (Mean values with their standard errors)

	Diets												Model	*R* ^2^	*P*		
	T + M 1		T + M 4		T + M 7		T + M 8		T + M 11		T + M 14				T	M	T * M
	Mean	se	Mean	se	Mean	se	Mean	se	Mean	se	Mean	se					
Dietary taurine and methionine contents (g/kg diet)																	
T^1^	1·6		11·9		20·4		1·6		11·7		20·0		–		–	–	–
M	12·0		10·8		11·1		16·7		18·8		15·0		–		–	–	–
C	6·8		5·4		5·7		5·9		5·2		5·8		–		–	–	–
Goblet cell and vacuole count per villus																	
TGC	93·8	5·1^a^	79·8	4·8^a^	117·4	6·5^a,b^	130·9	7^a,b^	108·5	6·3^a,c^	132·9	10·2^a^	M2^23^	0·55	***	NS	**
AB+	72·8	4·1^a^	54·2	3·1^a^	58·5	3·9^b^	55·2	3·5^b^	52·7	3·3^b^	62·2	5^b^	M2	0·39	***	***	***
PAS+	3·1	0·4^a^	4·8	0·6^a,b^	14·1	1·7^d^	11·5	0·9^c,d^	7·7	0·9^b,c^	10·5	0·9^c,d^	M1	0·11	***	***	***
AB + PAS+	17·8	1·6^a^	50·7	2·9^a,b^	44·8	4·0^b^	64·3	3·5^c^	48·1	4^b^	60·1	6·5^b,c^	M1	0·32	**	***	***
S-PAS+	106·2	11·5^a^	118·0	7·4^a^	107·4	8·5^a^	224·0	12·4^c^	185·4	11·9^b,c^	169·1	13·4^b,c^	M2	0·27	***	***	***
SV	66·8	3·9^a^	23·7	3·9^b,c^	15·8	3·9^c^	21·2	3·9^c^	18·7	3·9^c^	37·2	4·2^b^	M1	0·06	***	***	***
Density of goblet cell mucin and vacuoles/villus mm^2^																	
AB:PAS ratio	23:1		11:1		4:1		5:1		7:1		6:1		–	–	–	–	–
AB+/VA	0·059	0·002^a^	0·058	0·003^a^	0·038	0·001^b^	0·035	0·002^b^	0·031	0·001^b^	0·035	0·002^b^	M1	–	**	**	***
PAS+/VA	0·002	0·001^a^	0·005	0·001^a,b^	0·013	0·002^c^	0·007	0·000^b^	0·004	0·000^a,b^	0·006	0·00^a,b^	M1	–	***	***	***
AB + PAS+/VA	0·014	0·001^a^	0·022	0·005^a,b^	0·039	0·005^c,d^	0·041	0·002^d^	0·027	0·002^a,b,c^	0·033	0·002^b,c,d^	M1	–	***	***	***
TGC/VA	0·076	0·002^a,b^	0·085	0·006^b^	0·091	0·007^b^	0·084	0·003^b^	0·063	0·002^a^	0·074	0·003^a,b^	M1	–	NS	**	**
S-PAS+/VA	0·080	0·005^a^	0·125	0·005^b,c^	0·070	0·005^a^	0·150	0·007^c^	0·113	0·005^b^	0·107	0·010^b^	M1	–	***	NS	***
SV/VA	0·055	0·004^a^	0·021	0·003^b^	0·010	0·002^c^	0·013	0·002^b,c^	0·011	0·002^b,c^	0·018	0·002^b,c^	M1	–	***	***	***

T, taurine; M, methionine; C, cysteine; AB+, AB+ goblet cell mucus count; PAS+, PAS+ goblet cell mucus count; AB + PAS+, AB + PAS+ goblet cell mucus count; TGC, Total goblet cell mucus count, S-PAS+, Small PAS+ bullet-shaped bodies; SV, supranuclear vacuoles; VA, villus area.

The significant effects were determined by two-way ANOVA (M1) or ANCOVA (M2) and levels of significance are with respect to

*** *P* < 0·001.

** *P* < 0·01 and

^a,b,c^Data with the same superscript letter within the T + M rows are statistically similar (*P* < 0·05).

For the two-way ANOVA. TGC, AB+ and S-PAS+ in section ‘Goblet cell and vacuole count per villus’ were controlled for villus area after meeting assumptions; however, reported values are expressed as uncorrected mean values with their standard errors.

Besides larger goblet cells that stained PAS+ (41·4 (se 1·45) µm), there were also smaller bullet-shaped S-PAS+ bodies situated in the apical areas of the enterocytes with an average size of 18·36 (se 0·68) µm (Fig. 3(d)). The density of S-PAS+ was significantly affected by dietary Tau, which interacted with dietary Met and was the highest in YTK fed the T + M 8 diet (0·15 (se 0·007) per µm^2^). YTK had supranuclear vacuoles that were also situated in the apical cytoplasm of enterocytes in the lamina epithelial and were on average 21·4 (se 1·08) µm. The supranuclear vacuoles density in YTK fed the T + M diets was significantly affected by interacting dietary Tau and Met, where fish fed the T + M 1 diet exhibited an exceptionally higher supranuclear vacuole density (0·055 (se 0·004) per µm^2^) than other T + M diets.

### MetCys study and Com: posterior intestine

Overall, the posterior intestines appeared clinically inconspicuous. The posterior intestinal wall of YTK (140–230 g) was between 1 and 1·3 mm thick (Table 8). The microscopic observations and measurements revealed that the intestinal wall composition and thickness were similar to the samples collected from the TauMet study (MI: 0·4–0·5 mm; SC: 0·3–0·4 mm; LPA: 184–261/mm^2^). Each intestinal cross-section (4 µm) contained between 64 and 124 villi (total villi count) that ranged from 4·1 to 4·9 mm in length (VL) and exhibited multiple tips, which contributes to a greater complexity and surface area.

**Table 8.** Macro- and histomorphometric features of juvenile yellowtail kingfish (*Seriola lalandi*) posterior intestine fed one of four different methionine-cysteine levels from the MetCys study and the commercial diet. M + C 2 was closest in meeting the average MOM (14·3 g/kg diet) a and the TSAA (Met) requirement (26·2 g/kg diet) at 5·9 g Cys/kg diet in YTK^(2)^ (Mean values with their standard errors)

	Commercial diet (Com)				Experimental diets								Model	*R* ^2^	*P*		
	Initial		Final		M + C 1		M + C 2		M + C 3		M + C 4				M	C	M * C
	Mean	se	Mean	se	Mean	se	Mean	se	Mean	se	Mean	se					
Dietary methionine and cysteine contents (g/kg)																	
M^1^	12·6		12·6		8·8		24·7		7·9		25·2		–	–	–	–	–
C	6·5		6·5		5·8		5·9		13·7		13·9		–	–	–	–	–
T	7·5		7·5		7·0		6·9		7·0		6·9		–	–	–	–	–
Macromorphometrics (g, unless otherwise stated)																	
BW	52·7	0	327·2	4·3	246·3	7·7	254·1	8·9	257·6	3·4	256·2	8·3	–	–	NS	NS	NS
VW	NA	NA	NA	NA	14·3	0·5	14·6	0·6	14·2	0·1	15·1	0·6	–	–	NS	NS	NS
VSI (%)	NA	NA	NA	NA	6·1	0·1^a^	6·0	0·1^a^	5·5	0·0^b^	5·1	0·1^b^	–	–	NS	***	NS
LW	NA	NA	NA	NA	2·05	0·08	2·04	0·08	2·04	0·02	2·41	0·14	–	–	NS	NS	NS
HSI (%)	NA	NA	NA	NA	0·85	0·01	0·93	0·04	0·77	0·01	0·80	0·01	–	–	NS	NS	NS
Histomorphometry of posterior intestine (µm, unless otherwise stated)																	
PIC	28 551	654	45 361	803	39 769	831	47 620	826	4173	1544	44 131	871	M1	0·06	NS	NS	NS
TVH	2924	97	4755	174	5092	160^a^	4910	146^a^	4722	178^b^	4416	106^b^	M1	0·04	NS	**	NS
VL	2670	173	4106	184	4153	156^a^	4912	243^b^	4114	179^a,b^	4134	194^a,b^	M1	0·01	*	*	NS
TIW	716	13	1310	48	1320	37	1190	19	1159	42	1025	14	M1	0·01	NS	NS	NS
MI	261	5	472	23	516	15	540	24	439	20	387	4	M1	0·01	NS	NS	NS
S	275	4	357	20	450	11	337	8	388	16	422	12	M1	0·04	NS	NS	NS
TV	66	1	102	1	83	1	110	1	98	3	103	2	M1	0·02	*	NS	NS
VA (mm^2^)	693	50	1464	109	1316	78^a^	1461	107^b^	1143	72^b^	1155	71^c^	M1	0·04	***	***	NS
LEA (mm^2^)	567	43	1216	93	1094	65	1200	88	924	59	971	61	M2	0·99	NS	NS	NS
LPA (mm^2^)	126	9	248	19	222	16	261	20	219	15	184	12	M2	0·81	NS	NS	NS

M, methionine; C, cysteine; T, taurine; BW, total fish weight; VW, viscera weight; VSI, viscerosomatic index; LW, liver weight; HSI, hepatosomatic index; PIC, posterior intestine circumference; TVH, total villus height; VL, villus length; TIW, total intestinal wall thickness; MI, muscularis interna thickness; S, submucosa.

The significant effects were determined by two-way ANOVA (M1) or ANCOVA (M2) and levels of significance are with respect to

* *P* < 0·05,

** *P* < 0·01, and

*** *P* < 0·001.

^a,b,c^Data with the same superscript letter within the M + C rows are statistically similar (*P* < 0·05).

For the two-way ANOVA, LPA and LEA were controlled via ANCOVA for villus area after meeting assumptions; however, reported values were reported as uncontrolled mean values with their standard errors.

Histomorphometric measurements from the MetCys study were tested for possible effects of body weight and VA before being subjected to two-way ANOVA to test for Met and Cys effects on the dependent variables. However, relationships mostly violated linearity and were not statistically significant; thus, data were not controlled. Exceptions were the lamina epithelial and LPA, which strongly correlated with the VA and met assumptions to adjust means for VA via two-way ANCOVA.

YTK fed the M + C 2 diet, which was closest to meeting the average minimum obligatory methionine requirement (14·3 g/kg) and the TSAA (Met) requirement (26·2 g/kg) at 5·9 g Cys/kg in YTK^(2)^, had the longest villi, thickest MI, more villi in the posterior intestine and increased lamina epithelial area, indicating more complex villi and greater absorptive surface area. In comparison, YTK fed diet M + C 1, in which Met and Cys content were below that of the TSAA and MOM requirement, had the least villi in the posterior intestine, less lamina epithelial area and shorter villi, yet the thickest intestinal wall, whereas YTK that were fed the high dietary Cys series, M + C 3 and 4, had significantly shorter villi, decreased villi height, thinner intestinal walls, the least lamina epithelial surface and VA.

YTK sampled at the start of the MetCys feeding trial had substantially thinner PIC (28 551 (se 654) µm), TIW (716 (se 13) µm), MI (261 (se 5) µm) and S (275 (se 5) µm). YTK fed the Com or M + C diets and sampled after 8 weeks had comparatively thicker PIC, TIW, MI, stratum granulosum and stratum compactum indicating histomorphometric changes in the posterior intestine that may have been induced substantially through growth and ontogenic changes.

**Fig. 9.**
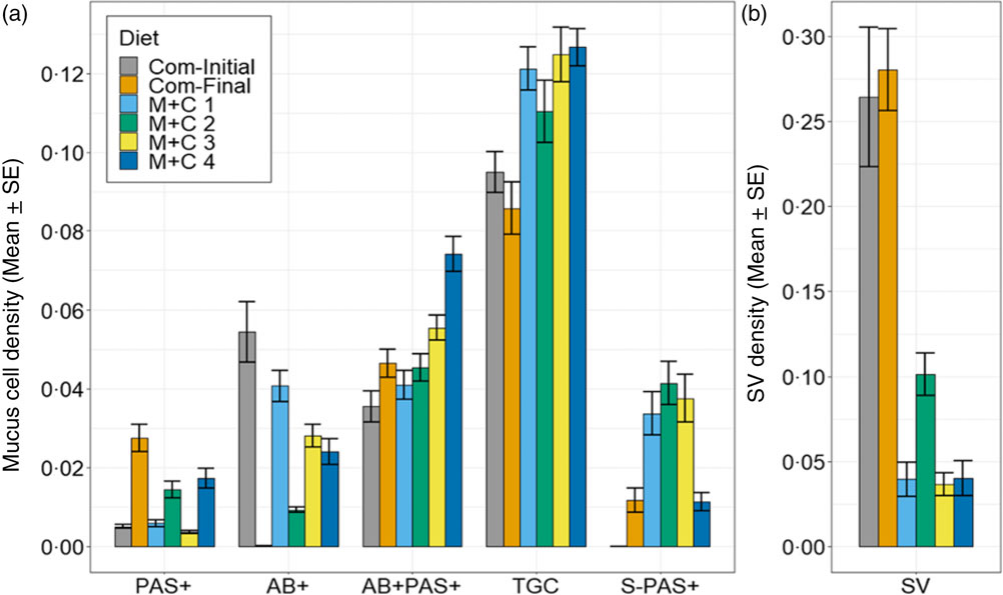
Barplots on the histochemical analysis of juvenile yellowtail kingfish (Seriola lalandi) posterior intestine, fed one of five methionine-cysteine combinations (MetCys). (a) Neutral goblet cell mucus (PAS+ per villus area), acid goblet cell mucus (AB+ per villus area), or mixed goblet cell mucus (AB + PAS+ per villus area), total goblet cell mucus (TGC per villus area) and bullet-shaped PAS+ mucus (S-PAS+ per villus area). (b) Supranuclear vacuole density (SV per villus area). Error bars indicate standard error.

The total goblet cell mucus production was most pronounced in fish fed the M + C 4 diet, exhibiting elevated goblet cell mucus densities of total goblet cells (TGC/VA) (Fig. 9). The histochemical analysis of the posterior intestine indicated three types of goblet cell mucus: acidic, neutral and mixed (Table 9). Goblet cell mucus cells predominately stained acidic (14–44 per villus) or mixed (56–78 per villus), whereas only a few goblet cell mucus cells stained neutral (TauMet study: 3–14 per villus; MetCys study: 4–16 per villus). YTK fed more dietary Met, and Cys exhibited more mixed and less acidic goblet cell mucus cells. YTK fed M + C, and Com diets also had supranuclear vacuoles and S-PAS+ bodies. YTK fed Cys below 13·9 g/kg but with higher Met concentrations had the highest supranuclear vacuoles density of YTK (diet M + C 2) fed the M + C diets (0·101 ± 0·012). However, the initial and final supranuclear vacuoles densities of YTK fed the Com diet were even more pronounced (0·280 (se 0·022) per µm^2^ and 0·264 (se 0·041) per µm^2^). Overall, YTK fed M + C and commercial diets exhibited considerably lower S-PAS+ and higher supranuclear vacuoles densities than YTK fed the T + M diets (Table 9).

**Table 9.** Histochemical analysis of mucus and supranuclear vacuoles in juvenile yellowtail kingfish (*Seriola lalandi*) intestine, fed one of six methionine-cysteine combinations (MetCys study) or a commercial diet. M + C 2 was closest to meeting the average MOM (14·3 g/kg diet) and the TSAA (Met) requirement (26·2 g/kg diet) at 5·9 g Cys/kg diet in YTK^(2)^ (Mean values with their standard errors)

	Commercial diet				Experimental diets								Model	*R* ^2^	*P*		
	Initial		Final		M + C 1		M + C 2		M + C 3		M + C 4				M	C	M * C
	Mean	se	Mean	se	Mean	se	Mean	se	Mean	se	Mean	se					
Dietary methionine and cysteine contents (g/kg diet)																	
M^1^	12·6		12·6		8·8		24·7		7·9		25·2		–	–	–	–	–
C	6·5		6·5		5·8		5·9		13·7		13·9		–	–	–	–	–
T	7·5		7·5		7·0		6·9		7·0		6·9		–	–	–	–	–
Goblet cell and vacuole count per villus																	
TGC	63·1	5·9	82·2	5·2	109·7	7·3^a^	102·2	10·1^a^	93·4	5·7^a^	121·2	7·5^b^	M2	0·47	NS	**	***
AB+	23·9	3·3	0·0	0·0	44·3	4·1^a^	14·0	1·5^a^	23·8	2·2^a^	27·5	4·2^b^	M1	0·05	*	NS	NS
PAS+	3·0	0·4	27·2	2·5	9·7	1·8^a^	22·7	4·0^b^	4·3	0·4^a^	15·9	2·2^b^	M2	0·40	***	NS	NS
AB + PAS+	23·9	3·3	55·0	3·8	55·8	5·9^a^	65·5	6·3^a,b^	65·3	5·4^b,c^	77·7	5·1^c^	M2	0·39	**	***	NS
S-PAS+	0·0	0·0	18·2	4·8	54·5	10·1^a^	65·3	11·1^b^	49·8	8·1^a,b^	17·5	4·1^a,b^	M2	0·35	NS	NS	**
SV	176·7	19·3	276·8	29·9	48·6	12·0^a^	165·8	26·6^b^	53·5	10·1^a^	56·1	15·0^a^	M1	0·27	***	**	***
Density of goblet cell mucin and vacuoles/villus mm^2^																	
AB: PAS ratio	8:1		0:1		5:1		1:1·6		6:1		2:1		–	–	–	–	–
TGC	0·095	0·005	0·074	0·006	0·087	0·003^a^	0·069	0·005^b^	0·087	0·003^a^	0·115	0·006^c^	M1	–	NS	**	**
AB+	0·054	0·007	0·000	0·000	0·041	0·040^a^	0·009	0·001^c^	0·028	0·003^b^	0·024	0·003^b^	M1	–	***	NS	***
PAS+	0·005	0·000	0·027	0·004	0·006	0·001^a^	0·014	0·002^b^	0·004	0·000^a^	0·017	0·002^b^	M1	–	***	NS	NS
AB + PAS+	0·036	0·004	0·046	0·004	0·041	0·004^a^	0·045	0·003^a,b^	0·056	0·003^b^	0·074	0·004^c^	M1	–	**	***	NS
S-PAS+	0·000	0·000	0·012	0·003	0·034	0·005^a^	0·041	0·006^a^	0·038	0·006^a^	0·011	0·002^b^	M1	–	NS	**	***
SV	0·280	0·022	0·264	0·041	0·039	0·010^a^	0·101	0·012^b^	0·036	0·007^a^	0·040	0·010^a^	M1	–	**	**	**

M, methionine; C, cysteine; T, taurine; AB+, AB+ goblet cell mucus count; PAS+, PAS+ goblet cell mucus count; AB + PAS+, AB + PAS+ goblet cell mucus count; TGC, total goblet cell mucus count, S-PAS+, small PAS+ bullet-shaped bodies; SV, supranuclear vacuoles; VA, villus area.

Levels of significance are with respect to

* *P* < 0·05,

** *P* < 0·01 and

*** *P* < 0·001.

^a,b,c^Data with the same superscript letter within the M + C rows are statistically similar (*P* < 0·05).

The significant effects were determined by two-way ANOVA (M1) or ANCOVA (M2).

For the two-way ANOVA, TGC, PAS+, AB + PAS+ and S-PAS+ were priorly controlled via ANCOVA for villus area after meeting assumptions; however, values were reported as uncontrolled mean values with their standard errors (*n* 6).

## Discussion

The TSAA, minimum obligatory Met and Tau requirements of juvenile YTK have recently been quantified based on indices such as optimised growth, feed efficiency and protein retention^(2,19)^. However, information on the liver and posterior intestine condition of juvenile YTK fed sub-optimal, optimal and supra-optimal combinations of Met, Cys and Tau remains unknown. This study provides primary data on the Met, Cys and Tau altering effects on the plasma biochemistry and functional and structural properties of hepatic and intestinal tissues, demonstrating that adequate intakes of Met, Cys and Tau can improve nutrient assimilation capacity and help to maintain homoeostasis. Diets T + M 11^(19)^ and M + C 2^(2)^ came closest to the Met, Cys and Tau specifications to meet YTK requirements for optimised growth, feed efficiency and protein retention^(2,19)^. As a reference, a level of 24·5 g Met+Cys/kg was recommended for YTK diets, provided that 7·7 g Tau/kg, at least 14·3 g Met/kg and no more than 13·9 g Cys/kg were supplied^(2,19)^.

### Liver morpho-histology

To our knowledge, this is the first study to observe that higher levels of dietary Tau led to significantly thinner TDW in fish. In mice and humans, intrahepatic TDW thickening and proliferation are a negative property that is linked to bile acid-induced inflammation, fibrosis and necrosis of cholangiocytes and hepatocytes with consequential reductions in biliary flow^(41,42)^. Tau may have alleviated the symptoms of BDW thickening by conjugating with hydrophilic bile acids that are less cytotoxic and that stimulate secretin hydrocholeresis^(43–46)^. Dietary Met supplementation may have increased the production of S-adenosyl Met, known to improve conditions of chronic liver diseases, and Tau^(47,48)^.

### Plasma biochemistry

Blood plasma biochemistry was compared with results from reference ranges on sick, subclinical and healthy YTK plasma biochemistry^(4)^. Biochemical blood plasma parameters were dependent on diet-induced Tau and Met changes, which were slightly either increased or decreased when fed sub-optimal levels of dietary Met and Tau compared with the reference ranges of healthy YTK. Overall, blood plasma lipid concentrations were up to 4 times lower than the suggested reference ranges for YTK (cholesterol: 5·4–8·5 mmol/l, TAG: 2·7–4·8 mmol/l), (F. Stephens, unpub. data) and Japanese yellowtail (cholesterol > 6·3 mmol/l, TAG > 3·7 mmol/l) (M. Maita, personal communication, 2012). However, the deviation from the latter communications may be due to differences in fat contents of fed diets, fish size or other factors. YTK fed dietary Met at 17·2 g/kg showed increases in plasma TAG contents, which is coherent with results reported for rats, where Met restrictions altered lipogenesis and decreased circulating lipid levels^(49)^. Additionally, YTK fed diets containing Met levels at 17·2 g/kg exhibited higher AST and LD activity, which are pathological markers for cell damage when exceeding reference range thresholds and correlate with findings of increased necrotic hepatocytes. However, in the absence of reference ranges or indication of pathology, elevated levels of these enzymes may indicate their activity level in the plasma rather than liberation from cell lysis. This is consistent with Liu *et al.* ^(50)^, who found elevated LD for the fastest growing YTK in their study, consistent with its role in energetics. Likewise, the elevated AST levels for fish fed higher levels of Met found in our study are likely due to altered amino acid metabolism rather than pathology. LD is a glycolytic enzyme critical for fish burst swimming capacity and directly linked to metabolic rate^(51)^. Met is metabolised to cystathionine, which can form *α*-ketoglutarate, which is transaminated by AST to glutamate^(52)^.

### Liver surface colour

Schmitt and Dethloff^(53)^ subjectively described the colour of ‘normal’ liver as red to light red and fatty liver as ‘coffee with cream’ in largemouth bass (*Micropterus salmoides*) and redbreast sunfish (*Lepomis auritus*). In contrast, Brusle and Anadon^(54)^ described the colour of ‘normal’ fish liver as red–brown and fatty liver as yellow. The liver surface colour of YTK fed the T + M diets was bluer, greener and 16 % lighter than the hex colour ‘dark red’ (#AB2328), which is subjectively classified as a ‘normal’ liver by Brusle and Anadon^(54)^ and Schmitt and Dethloff^(53)^.

Previously, the subjective assessment of zebrafish linked liver surface colour lightness to hepatic TAG accumulations^(55)^. The digital assessment of broiler liver surface colour linked lightness to a carbohydrate-rich diet, whereas a darkening was linked to feeding less fat, more Met or fasting animals for 12 h^(36)^. YTK from this study had more cholesterol and TAG in blood plasma when fed the higher Met diets (17·2 g/kg) but interestingly not a higher fattiness score in their liver. The subjective and semi-quantitative hepatic fattiness scoring may not have been sensitive enough to capture dietary Met effects^(56)^; however, hepatic fat positively correlated with the yellow–blue channel (b*), indicating that increased hepatic fat made the liver surface colour appear more yellow.

### Relationship of intrahepatic bile duct walls and liver surface colour

Interestingly, the intrahepatic BDW thickness correlated with the liver surface colour, where thickness was significantly affected by dietary Tau. YTK fed T + M 7 and 14, which contained the maximum inclusion of dietary Tau (20·4 and 20·0 g/kg) and YTK fed T + M 1, which contained the least amount of dietary Tau, had the thinnest BDW. Yet only YTK fed T + M indicated the least red and bright livers (hex colour: B86948). Bile ducts are responsible for the transport of bile, which is comprised of water, bile salt, conjugated bilirubin (yellow), cholesterol, lecithin and inorganic substances from the hepatocytes into the intestine for digestion and waste removal^(54,57)^. A thickening of the intrahepatic TDW may indicate a compromised export of breakdown and bile products and may consequently alter the liver surface colour. Further research is warranted, but to our knowledge, the present study is the first to establish a link between liver surface colour and Tau-dependent BDW thickness. The liver surface colours of YTK fed diets T + M 3 and T + M 4, which contained sufficient dietary Tau to meet the requirement at a Met content of 11 g/kg, were up to 21 % more red (a* = 34·2 and 34·0) than YTK fed any other T + M diet. YTK fed the T + M 11 diet, which was closest to meeting the TSAA requirement and met the Tau requirement, had the reddest yet brightest liver surface colour throughout all treatments. This suggests that YTK fed dietary Tau and Met close to the requirement had better liver conditions, assuming that redder livers are necessarily healthier livers. The accuracy of a subjective colour scoring system for fish liver remains questionable: firstly, because relationships between objective, quantitative histology and macroscopic colour scores have not been established, and secondly because the subjective judgement relies on the observer’s perception of colour. At this stage, subjective colour assessments may serve as a simple clinical tool when underlying health conditions present in strong colour variations. More subtle colour changes may not be visible to the naked eye, whereas standardised instruments may overcome this issue.

### Green liver syndrome

In our study, YTK livers did not exhibit segmental or diffuse green discolourations as previously described by Bowyer *et al.* ^(58)^ in YTK and Takagi *et al.* ^(59)^ in red seabream. Japanese Yellowtail, red seabream (*Pagrus major*), and Totoaba (*Totoaba macdonaldi*) fed low-fishmeal diets deficient in Tau exhibited accumulated biliverdin and bilirubin in the liver due to increased haemolysis and insufficient conjugation with Tau for the excretion into the biliary system^(22,23,27,59–61)^. However, YTK fed a Tau and Met deficient diet (T + M 1 diet) had no signs of green liver discolourations, indicating that Tau deficiencies may not cause green liver syndrome but might exacerbate previous conditions. This finding is similar to YTK that were fed soya protein concentrate based diets low in Tau, Met and lysine, yet had no green liver^(62)^, whereas YTK fed a 100 % rapeseed oil-based diet and reared at 18°C had green livers. However, dietary Tau content in the experimental diets was not presented in that study^(63)^. Interestingly, in the cases where low Tau correlated with green liver, the fish were often exposed to low temperatures (12·3–18·0°C) and/or fed alternative protein sources, indicating that low water temperature may contribute to green liver^(64)^, including in Japanese yellowtail^(27)^, red seabream^(22)^ and YTK^(58)^. In poikilothermic frogs and salmon, hyperthermia increases haemolysis, increasing the biliverdin production, yet it reduces the biliverdin reductase, decreasing biliverdin (green) removal^(65)^. This may lead to an accumulation of hepatic biliverdin and subsequently a green discolouration. YTK from this study were held at 23·3 (se 0·6)°C, which may be within the thermal tolerance to maintain a healthy balance between production and removal of haematogenous pigments. Japanese yellowtail held at 23·9 (se 2·0)°C and fed non-fishmeal diets had green liver due to mucosporozoa infestations that blocked the biliary system, indicating that the diet possibly made the fish susceptible to infestation^(66)^. Overall, it appears that the green liver condition in fish may be induced by distinct physiological mechanisms that are triggered by a single factor or an interplay of factors such as imbalanced diet, toxicity, pathogens and water temperature.

### Posterior intestinal wall

Although it is widely recognised that dietary Tau and Met supplementation may be of benefit for fish health and growth^(5,67,68)^, in this study the submucosa of YTK was not affected by either dietary Met or Tau. Adequate dietary Met can down-regulate pro-inflammatory and up-regulate anti-inflammatory cytokine mRNA levels^(69)^. In Atlantic salmon, soya protein-based diets induced inflammatory responses and consequently increased stratum compactum/submucosal thickness^(70)^. Interestingly, with an incremental increase in both dietary Met and Tau, the unaffected submucosa was reflected by a marked expansion rather than a reduction in the lamina propria. This observation is different from what one would expect. Expansion of the submucosa and lamina propria by immune cell infiltration usually coincides^(71–73)^, but the expansion may also result from the lamina propria’s and stratum granulosum’s division by the stratum compactum, about which immune cell exchange is little known.

Moreover, it is possible that the observed expansion of the lamina propria is not the product of an increased immune cell infiltration but may also be related to its additional role as circulatory system for nutrients and blood^(74)^. Overall, adequate dietary Tau and Met left the submucosa unaffected, yet simultaneously not the lamina propria. The reason behind this effect is unclear, and further research is warranted to understand the intestinal immune cell exchange and infiltration.

### Posterior intestinal mucus production and composition

Goblet cell mucus is divided into two pH types. Acidic mucus (AB+/blue) is suggested to form a barrier against pathogens, resisting degradation of bacterial glycosidase activity and limiting bacterial translocation by increasing viscoelasticity. Neutral mucus (PAS+/magenta) forms a physical barrier that promotes digestion and neutralises digestive juices, protecting the lamina epithelial from autodigestion^(75–77)^. The posterior intestine is often exposed to a higher bacterial load and has been shown to contain more acidic goblet cell mucus that is protective against pathogens^(75,78)^. However, lipids and proteinogenic amino acids may be unavailable for absorption until reaching the posterior intestine^(79)^ and therefore, the posterior intestine may, to a certain extent, have goblet cell mucus that assists with the nutrient assimilation.

YTK from our study had predominantly acidic and mixed posterior intestine goblet cell mucus and fewer neutral goblet cells, indicating that properties of the goblet cell mucus were protecting the system from pathogens. However, an increase of dietary Met, Cys or Tau decreased the secretion of acid goblet cell mucus and simultaneously stimulated the production of mixed and neutral goblet cell mucus, shifting the properties towards more digestive and absorptive functions. In a previous study, YTK had more neutral goblet cell mucus, in which composition was unaffected by soya protein inclusions and water temperature^(80)^. However, unlike our study, that study defined neutral and mixed goblet cells mucus as neutral^(80)^. The anterior intestine of uninfected brown trout (*Salmo trutta*) had predominately neutral goblet cell mucus, whereas *Cyathocephalus truncates*-infected brown trout had 4 times more acid goblet cell mucus (43/villus) than neutral goblet cell mucus (10/villus), which is similar to the YTK from our study exhibiting 2–27 times more acid than neutral goblet cell mucus. This highlights the importance of acid goblet cell mucus as an immune response to pathogens in the posterior intestine of fish.

Apart from shifting the pH of the goblet cell mucus composition into the acidic range, the posterior intestine can also temporarily increase mucus secretion upon bacterial recognition^(81)^, acting as a lubricant and attacking pathogens^(82–84)^. Yet, it has been suggested that less goblet cell mucus improves nutrient absorption capacity^(77)^. YTK fed the T + M diets had 94–133 TGC per villus, which is similar to the TGC of the anterior intestine of *Pomphorhynchus laevis* infected brown trout (110 TGC per villus)^(85)^. YTK fed the T + M 11 and M + C 2 diet, which nearly met dietary Met, Cys and Tau requirements^(2,19)^, had the least number of total goblet cells indicating that Met, Cys and Tau complete diets lessen the total goblet cell mucus production and improve absorption capacity. Overall, YTK indicated a shift towards absorptive properties when fed diets that contained adequate dietary Met, Cys and Tau levels.

### Posterior intestinal surface area

Enterocytes within the lamina epithelia are cells that absorb amino acids, simple peptides, lipids and monosaccharides via endocytosis, catabolise nutrients and release them into the circulation^(86)^. In YTK, amino acid retention was shown to depend on the water temperature and dissolved oxygen concentrations^(87)^. Intestinal characteristics associated with increased absorptive area include enterocyte density, total villus height, VL, lamina epithelial area, villus density and intestinal circumference. YTK fed increasing dietary Met and Tau exhibited an increase in absorptive surface area. Further, YTK exhibited a decreasing absorptive surface area when fed Cys at 13·9 g/kg, marking an upper Cys threshold, which agrees with the suppressed growth and feed efficiency observed in YTK fed the same dietary Cys contents^(2)^. Overall, YTK fed dietary Cys below 13·9 g/kg had more villi in the posterior intestine and an increased lamina epithelial area, thus larger surface areas for the absorption of nutrients.

YTK had large, transparent supranuclear vacuoles in the cytoplasm of enterocytes. These vacuoles are presumed to have contained lipids that were washed out during histological processing as they did not stain positive for glycogen (PAS+) or protein (eosin). Deficiency in both Tau and Met (T + M 1) caused increased vacuolisation in the posterior intestine tissue and may be caused by increased lipid absorption or decreased clearance from enterocytes into blood circulation^(88)^. Similar results have been attributed to lipid-rich diets^(89)^, deficiencies of some fatty acids^(90)^ or diets rich in soyabean proteins^(73,91)^. Since plasma TAG levels were particularly low for fish fed the low Met and low Tau diet, this result indicates that diets low in Tau and Met concurrently decrease the clearance of lipids from intestinal enterocytes and thereby decrease energy available for metabolism. YTK also had S-PAS+ bodies, indicative of glycogen or glycogen-rich accumulations such as lysosomes^(92)^. YTK fed the T + M diets, which contained 23·8 % carbohydrate, had substantially more 185 S-PAS+ bodies per villus in comparison with YTK fed the M + C diets, which contained only 12·3 % carbohydrates and induced only half as many S-PAS+. Thus, increased dietary starch may increase dietary glycogen deposition.

### Conclusion

To conclude, dietary Met, Cys and Tau-induced variations in the liver and posterior intestine suggest a shift in functional and structural properties. YTK fed insufficient dietary Met, Cys or Tau exhibited decreased red colouration in their livers, acidic goblet cell mucus with immune responsive properties, reduced absorptive surface area and increased accumulation of lipid supranuclear vacuoles. However, when YTK were fed diets containing Met, Cys and Tau close to their physiological requirements, TDW were relatively thinner, livers appeared redder and brighter, goblet cell mucus shifted towards absorptive properties, the intestinal absorptive surface area increased and YTK had less supranuclear vacuoles in villi enterocytes. These findings form a coherent, overarching picture with our previous findings of YTK fed dietary SAA and Tau at sub-optimal or supra-optimal levels, exhibiting inferior growth, feed efficiency, protein retention and eye health. This contrasts with YTK fed adequate levels and which subsequently exhibited a relatively superior response. Therefore, hepatic and intestinal histological responses that were statistically different to YTK fed adequate levels of Met, Cys and Tau may reflect nutritionally incomplete diets. With increasing commercial YTK aquaculture production, optimised gastrointestinal and liver function form critical components for good health and productivity. This study has shown that dietary SAA and Tau concentrations alter nutrient absorption and protective properties and provide insight into the macroscopy, histomorphology and histochemistry of the liver and posterior intestine. Further, this study provides useful information on methods for the collection of quantitative data from histological sections and liver surface colour rather than relying on subjective or qualitative evaluations and can be useful to implement deep learning algorithms that assist in automating this otherwise laborious process. Nevertheless, further research is needed to investigate the relationships between histology and organ function, particularly regarding intestinal goblet cell mucus, stratum granulosum and immune responses.
